# Sociodemographic inequalities in long-term exposure to air pollution, road traffic noise, and greenness: A population-based cohort study of women

**DOI:** 10.1097/EE9.0000000000000279

**Published:** 2023-12-01

**Authors:** Lara Stucki, Staffan Betnér, Jenny Selander, Mare Lõhmus, Agneta Åkesson, Charlotta Eriksson

**Affiliations:** a Institute of Environmental Medicine, Karolinska Institutet, Stockholm, Sweden; b Centre for Occupational and Environmental Medicine, Region Stockholm, Stockholm, Sweden

**Keywords:** Air pollution, PM_2.5_, PM_10_, NO_2_, Road traffic noise, Greenness, Green space, Sociodemographic inequalities, Lifestyle

## Abstract

**Background:**

Recent evidence suggests environmental health inequalities both within and between European countries and socially deprived groups may be more susceptible to pollution. However, evidence is still inconclusive and additional studies are warranted. This study aims to investigate sociodemographic inequalities in long-term residential exposure to air pollution, road traffic noise, and greenness, taking lifestyle and degree of urbanization into account.

**Methods:**

In total 20,407 women, born 1914–48 residing in Uppsala County, Sweden, were followed between 1997 and 2017. Time-varying sociodemographic variables were obtained from registers, and questionnaires provided lifestyle information. Generalized estimating equations were used to compute beta-coefficients (β) and 95% confidence intervals (95% CI) for associations between sociodemographic and lifestyle variables and spatial-temporal modeled particulate matter (PM_2.5_, PM_10_), nitrogen dioxide (NO_2_), road traffic noise and greenness. All models were additionally stratified by urbanization type.

**Results:**

Urban area residency was the most important predictor of high exposure to air pollution and noise, and to low greenness. For instance, β for NO_2_ was −2.92 (95% CI = −3.00, −2.83) and −3.10 (95% CI = −3.18, −3.01) µg/m^3^ in suburban and rural areas, respectively, compared with urban areas. For greenness, the opposite held true with corresponding β of 0.059 (95% CI = 0.056, 0.062) and 0.095 (95% CI = 0.092, 0.098). Within urban areas, elderly, unmarried and well-educated women had the highest environmental burden. However, less pronounced, and even reversed associations were found in suburban and rural areas.

**Conclusion:**

This study provides evidence of a mixed pattern of environmental health inequalities across sociodemographic groups in urban areas.

**What this study adds**

This study adds important scientific evidence of the sociodemographic drivers behind environmental health inequalities which is needed as a basis for preventive actions, policy decisions, and sustainable urban planning. The study clearly indicates a higher environmental burden among women living in urbanized areas, as compared with those living in suburban or rural areas, and points to a mixed pattern of environmental inequalities across different sociodemographic groups. Furthermore, the study highlights the need for detailed and location-specific knowledge of the occurrence and distribution of environmental exposures within the population as a basis for public health prevention.

## Introduction

Exposure to urban environmental factors such as air pollution and traffic noise have been related to a wide range of adverse health effects in the general population. Typically, air pollutants such as particles with an aerodynamic diameter of <2.5 µm (PM_2.5_) and <10 µm (PM_10_) and nitrogen dioxide (NO_2_) have been associated with impaired respiratory health, cardiovascular disease and lung cancer.^1–3^ Common disturbances in noise-exposed populations include general annoyance, impairments of rest and relaxation, and sleep deprivation.^4,5^ Mounting evidence also suggests an association between long-term exposure to road traffic noise and increased risk of ischemic heart disease.^6^ On the other hand, exposure to urban greenness has been suggested to promote health in various ways and to be related to overall lower mortality rates.^7^ The pathways between greenness exposure and health are thought to include reduced stress, increased physical activity, and a mitigating impact on the negative health effects of air pollution and traffic noise.^8^

Environmental health inequalities refer to a disproportionate or unfair distribution of environmental health hazards within the general population which implies that some groups are more exposed to environmental health risks than others.^9^ In conjunction with an increased susceptibility to poor health within these groups, often related to socioeconomic status (SES) and lifestyle, inequalities in exposure to environmental pollution may lead to population health disparities. Recent reviews point to inequalities in the exposure to environmental pollutants both within and between the European population where, in general, the most vulnerable social groups, poorer populations, and minorities are the most exposed and the most susceptible groups.^10,11^ However, the patterns are not entirely consistent, and increased knowledge that integrates the environmental and social domains and explores the drivers behind inequalities in environmental exposure and its potentially disproportionate health effects is greatly needed. For instance, information of this kind is of importance to tailor preventive actions for improving population health, as a basis for policy decisions, and for sustainable urban planning. Information about environmental health inequalities is also needed to better understand the role of sociodemographic determinants as predictors in environmental health research.

In this study, we aimed to investigate sociodemographic inequalities in long-term residential exposure to air pollution (particles and nitrogen dioxide), road traffic noise and greenness, respectively, within a population of female residents in Sweden. Furthermore, we also aimed to assess inequalities in environmental exposure according to individual lifestyle and to explore potential differences in associations according to degree of urbanization.

## Methods

### Study population

The study builds on prospective population-based data from the Swedish Mammography Cohort (SMC) (Figure S1; http://links.lww.com/EE/A250), previously described in Harris et al.^12^ In brief, the SMC was established between 1987 and 1990 when all women living in Uppsala and Västmanland counties, born 1914 to 1948, were invited to a mammography screening and questionnaire survey to which 66,651 women responded (74%). At recruitment, from 1987 to 1990, the SMC population was representative of the general female population in Sweden regarding the distribution of age, education level, and body mass index (BMI).^12^ As a baseline for the present study, we used data from a questionnaire survey performed within the SMC in 1997 (response rate 70%), including female residents from Uppsala County. Additional questionnaires were completed in 2008 and 2009, providing updated information on lifestyle and behavior. The women were followed for 20 years, that is, until the end of 2017, taking moving within the County of Uppsala into account using information from the population register held by the Swedish Tax Agency. During the study period, 6,478 participants died and 522 moved out of the study area. Additionally, 81 participants were excluded due to missing data on exposure and restriction to the study period. The final number at baseline (1997) comprised 20,244 women.

### Exposure assessment

The environmental exposures under study in this project were total concentrations of PM_2.5_, PM_10_, and NO_2_, road traffic noise and residential greenness. The participants' exposure to these factors was assessed at each address where they had lived during the study period by combining geographical address coordinates with exposure information using geographic information system technique. For each participant, we obtained detailed information on residential history for the study period from the Swedish Tax Agency. The addresses were geocoded by matching to the Real Property Register, held by the Swedish mapping, cadastral and land registration authority, and thereafter linked to individual-level exposure data.

Exposure to air pollution was assessed by dispersion modeling.^13^ Sources considered were emissions from road traffic (including street canyon effect where applicable), boilers and energy plants, individual heating with solid fuel (wood) and oil, shipping, and long-distance transport. Annual time-weighted average total concentrations of PM_2.5_, PM_10_, and NO_2_ were calculated for all study participants.

Exposure to road traffic noise was assessed using the Nordic prediction method.^14^ Input data included ground surface, road net and traffic flows on both state-owned and municipal roads, diurnal distributions, percentage of heavy vehicles, speed, and buildings. The exposure was calculated as free-field levels at the façade of buildings at 2 m height and are expressed as dB *L*_den_ which is the day-evening-night noise level over an entire day, imposing a penalty of 5 and 10 dB on sound levels during evenings and nights, respectively. *L*_den_, was calculated for every 5th year from 1990 to 2015. To interpolate annual noise exposure, we applied linear regression and calculated the annual time-weighted individual average.

Exposure to greenness was assessed from satellite images depicting the Normalized Difference Vegetation Index (NDVI) for Uppsala County.^15^ Overall, we obtained the maximal NDVI for each year (i.e., during the summer months) between 1995 and 2017, however, 9 years thereof were missing due to poor data quality. Missing values were replaced by the median of the four closest years available. Furthermore, we corrected the data for cloud contamination using a method previously applied in Stockholm County.^16^ Finally, annual time-weighted individual NDVI was assessed within a 500 m buffer around the women’s residential addresses.

### Sociodemographic determinants

Information on sociodemographic determinants of our participants throughout the study period was obtained from registers held by Statistics Sweden, including the Total Population Register, the People and Housing Census, the Longitudinal Integrated Database for Health Insurance and Labor Market Studies, and the Geodatabase. For the purposes of the present study, we selected a set of key sociodemographic variables of interest as outlined below and in Table 1.

**Table 1.** Characteristics of the study population.

Variable	1997	2008	2017
	n = 20,244	n = 17,018	n = 12,682
Age^a^ (years), median (Q1; Q3)			
	60.0 (53.0; 70.0)	70.0 (64.0; 79.0)	76.0 (72.0; 83.0)
Age^a^ tertiles, n (%)			
T1 (04/1942–12/1948)	6,737 (33.3)	6,161 (36.2)	5,605 (44.2)
T2 (02/1931–03/1942)	6,740 (33.3)	6,032 (35.5)	5,059 (39.9)
T3 (01/1914–01/1931)	6,761 (33.4)	4,819 (28.3)	2,012 (15.9)
Civil status^a^, n (%)			
Married or registered partner	12,875 (63.7)	8,882 (52.2)	5,634 (44.4)
Unmarried	1,472 (7.28)	1,089 (6.40)	820 (6.47)
Divorced or widowed	5,866 (29.0)	7,041 (41.4)	6,222 (49.1)
Employment status^a^, n (%)			
Gainfully employed	9,912 (49.6)	3,719 (21.9)	101 (0.80)
Not gainfully employed	2,004 (10.0)	1,013 (5.98)	0 (0.00)
Retired	8,080 (40.4)	12,218 (72.1)	12,575 (99.2)
Education^a^ (years of education), n (%)			
<10 (presecondary)	8,009 (39.7)	6,385 (37.6)	4,122 (32.5)
10–12 (high school)	6,735 (33.4)	5,822 (34.3)	4,526 (35.7)
>12 (postsecondary/postgraduate)	5,409 (26.8)	4,767 (28.1)	4,020 (31.7)
Disposable individual income^a^ (SEK), median (Q1; Q3)			
	106,500 (76,900; 144,000)	133,400 (105,300; 196,425)	152,700 (127,500; 193,000)
Disposable household income^a^ (SEK), median (Q1; Q3)			
	203,400 (135,400; 299,800)	238,700 (139,275; 371,525)	244,350 (153,700; 363,800)
Area-based (DeSO) income^a^ (SEK), median (Q1; Q3)			
	150,842 (140,138; 161,591)	210,554 (189,178; 225,723)	239,798 (220,770; 266,233)
Urbanization type (DeSO), n (%)			
Urban	12,931 (63.9)	11,202 (65.8)	8,647 (68.2)
Suburban	2,970 (14.7)	2,495 (14.7)	1,847 (14.6)
Rural	4,336 (21.4)	3,315 (19.5)	2,182 (17.2)
Alcohol consumption^b^ (g ethanol/day), median (Q1; Q3)			
	4.25 (5.36)	4.87 (5.65)^c^	–
Smoking^b^, n (%)			
Never	10,704 (54.1)	5,794 (58.4)^c^	–
Former	4,562 (23.1)	3,221 (32.5)^c^	–
Current	4,524 (22.9)	907 (9.14)^c^	–
Exercise^b^ (hours/week), n (%)			
<1	3,650 (20.4)	6,392 (61.7)^c^	–
1–3	10,333 (57.7)	3,763 (36.3)^c^	–
>3	3,916 (21.9)	209 (2.02)^c^	–
BMI^b^ (kg/m^2^), n (%)			
<25	11,390 (57.5)	6,001 (51.0)	–
25–30	6,414 (32.4)	4,112 (34.9)	–
>30	2,021 (10.2)	1,653 (14.0)	–

Population characteristics at baseline in 1997 and at follow-ups in 2008 and 2017.

a Register origin.

b Questionnaire origin.

c Data obtained from the questionnaire in 2009 instead of 2008.

DeSO indicates demographic statistical areas; SD, standard deviation; SEK, Swedish Kronor.

The selected individual sociodemographic variables included age (tertiles), civil status (married/registered partner, unmarried, and divorced/widowed), employment status (gainfully employed, unemployed, and retired), highest achieved educational level (presecondary education up to 9 years, high school education up to 3 years, postsecondary/postgraduate education), annual average disposable individual income (quartiles with cutoffs at baseline of 76,900, 106,500 and 144,100 SEK) and annual average disposable household income (quartiles with cutoffs at baseline of 135,375, 203,200, and 299,800 SEK).

The selected contextual variables were based on so-called demographic statistical areas (DeSO). DeSO divides Sweden into small areas with 700 to 2,700 inhabitants based on geographical boundaries (e.g., streets, railways, rivers, blocks, and electoral districts), aiming to capture small within-area and large between area socioeconomic variability. At this contextual (DeSO) level, we obtained the area-based income (quartiles with cutoffs at baseline of 140,138, 150,842, and 161,591 SEK) and urbanization type, categorized as: (1) rural, that is outside major population concentrations, (2) suburban, that is in a population concentrated area but not in the municipalities center, and (3) urban, that is in the municipalities central town.^17^ All sociodemographic variables were updated every year from 1990 to 2017 analogous to the time-varying exposure data and assigned on an individual basis to each study participant.

### Lifestyle variables

Information on lifestyle variables was obtained from the survey questionnaires in 1997 and in 2008/2009, respectively, and is thus updated only once during the study period. Selected lifestyle variables included alcohol consumption (in tertiles of g ethanol/day), smoking (never, former, and current), exercise (<1 hour/week, 1–3 hours/week, and >3 hours/week) and BMI (<25, 25–29, and ≥30 kg/m^2^).

### Statistical analyses

To describe the distribution of the sociodemographic and lifestyle variables in our population, we tabulated means and standard deviations for continuous variables, and numbers and percentages for the categorical variables at three different time points during the follow-up period: 1997, 2008/09, and 2017. To assess correlations between the continuous socioeconomic variables (i.e., individual, household, and area-based income), we computed Pearson correlation coefficients, for the baseline year (1997).

Time-trends in exposure to particles (PM_2.5_ and PM_10_), nitrogen dioxide (NO_2_), road traffic noise, and NDVI in our cohort were assessed by plotting the annual average exposure among the study participants from baseline (1997) to the end of follow-up (2017) for each environmental factor, respectively. In addition, we used overlaying frequency histograms to describe the distributions of the environmental factors in 1997, 2008, and 2017.

To explore sociodemographic inequalities in long-term residential exposure to environmental factors, we first plotted the annual average exposure among the study participants throughout the study period according to the categories of the selected sociodemographic and lifestyle variables. Next, we assessed associations between the sociodemographic variables (i.e., as independent variables) and the residential environmental exposures (i.e., as the dependent variable) using linear regression in generalized estimation equations (GEE)^18^ with a Gaussian family, an identity link function, and an autoregression correlation matrix structure (AR1) among repeated exposure occasion (i.e., 21 calendar years). Because NO_2_ appeared to have an exponential distribution (Figure S3; http://links.lww.com/EE/A250), we used the Gamma family with the inverse link function for this exposure. All models accounted for time-varying covariates, including age, civil status, employment status, education, individual income, household income, area-based income, and urbanization type as independent variables, categorized as described above. The results of the GEE model are presented as beta-coefficients (β) and 95% confidence intervals (95% CI) for PM_2.5_, PM_10_, NO_2_, road traffic noise, and NDVI, respectively.

To explore population inequalities in environmental exposure according to individual lifestyle, we additionally included the questionnaire-derived lifestyle variables (i.e., alcohol, smoking, exercise, and BMI) as predictors of exposure in the models. The lifestyle variables obtained in 1997 were used for the first half of the study period and once updated for the second half with the variables obtained from further questionnaires in 2008/09. Furthermore, to assess if the associations differed depending on urbanization type, we stratified our models according to DeSO-urbanization type, that is, in urban, suburban, and rural areas.

To implement GEE, we used the geeglm function from the geepack package.^19^ For data cleaning, management, and plotting we used packages within tidyverse,^20^ and for NDVI exposure assessment (geographic information system) we used the packages raster,^21^ sp,^22^ and sf^22^ in R (version 4.0.4; R Development Core Team).

The study was approved by the Regional Ethical Review Board in Stockholm (2018/1482-31) and was carried out according to the Code of Ethics of the World Medical Association (Declaration of Helsinki).

## Results

At baseline (1997), the median age of the 20,244 included women was 60 years, a majority were married (63.7%) and approximately half of them were still gainfully employed (49.6%) (Table 1). Most women lived in urban areas (63.9%), and 22.9% were current smokers. Compared with the baseline population, 84.1% and 62.6% were still alive and remained in the study area in 2008 and 2017, respectively. At the end of the follow-up, almost all women were retired (99.2%) and a slightly greater proportion lived in urban areas (68.2%). The highest correlation between the three continuous sociodemographic variables was found for individual and household income with *r* = 0.58 (Figure S2; http://links.lww.com/EE/A250).

**Figure 1.**
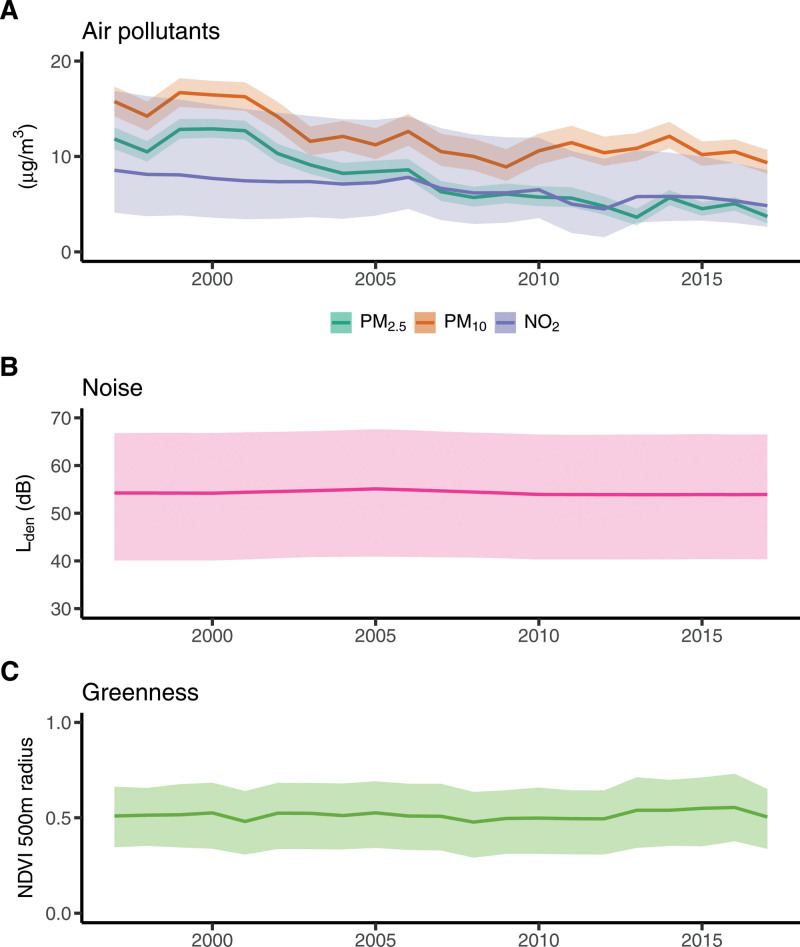
Time-trends of environmental exposures over the study period. Time-trends of the exposure to (A) air pollution (PM_10_, PM_2.5_, and NO_2_), (B) road traffic noise, and (C) greenness from baseline (1997) to end of follow-up (2017) among study participants of the Swedish Mammography Cohort (SMC) residing in Uppsala County, Sweden.

Median concentrations and their quartiles one and three of PM_2.5_, PM_10_, and NO_2_ at baseline were 11.9 (11.1; 12.4) µg/m^3^, 15.6 (14.7; 16.4), and (4.70; 10.6) µg/m^3^, respectively (Figure 1). Median level of road traffic noise was 55.4 (48.8; 59.8) dB *L*_den_, and the average NDVI within a 500 m buffer was 0.51 (0.45; 0.58). During the 20 years of follow-up, there was a significant decrease in the exposure to air pollution within the cohort, with median concentrations of 3.76 (3.48; 3.94), 9.32 (8.79; 9.67), and 3.89 (2.96; 5.63) µg/m^3^ for PM_2.5_, PM_10_, and NO_2_ respectively in 2017 (based on subjects remaining in the study). However, the average exposure to road traffic noise and NDVI remained virtually unchanged throughout the follow-up, with medians of 54.8 (48.3; 59.1) dB *L*_den_ and 0.53 (0.46; 0.58), respectively, in 2017.

**Figure 2.**
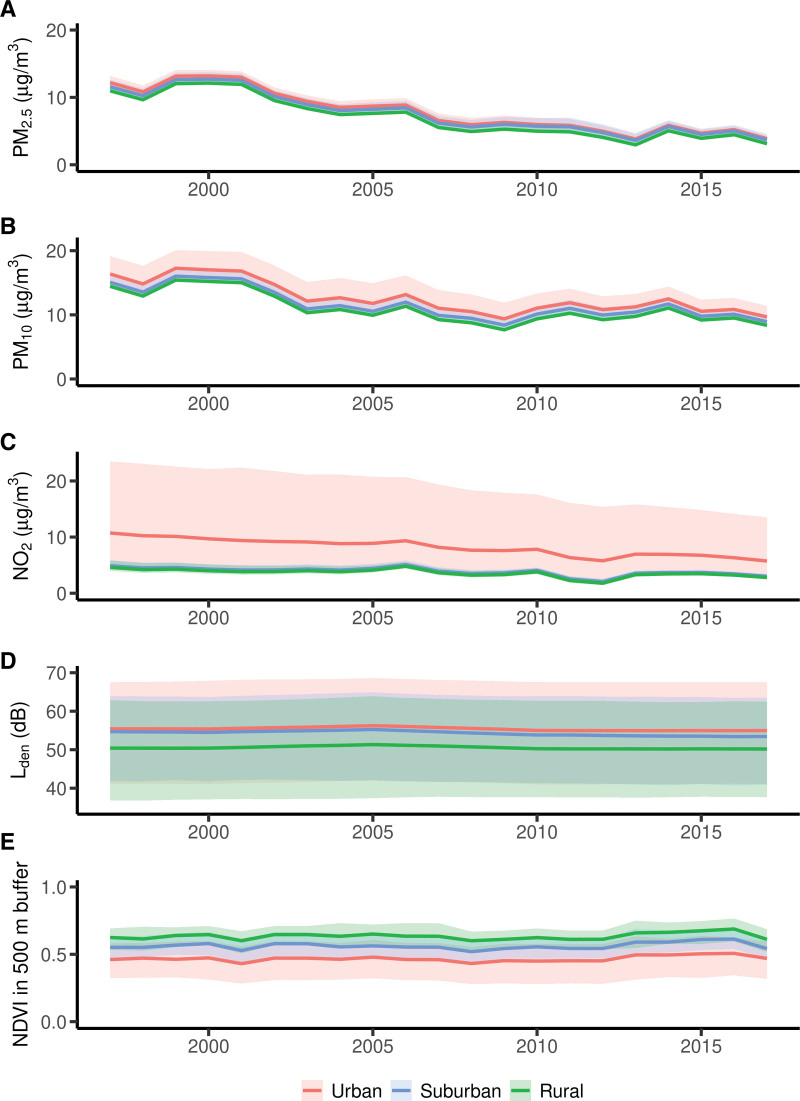
Time-trends of environmental exposures by residential urbanization type. Annual average and 5th and 95th percentile of the exposure to (A) PM_2.5_, (B) PM_10_, (C) NO_2_, (D) road traffic noise, and (E) greenness (500 m radius buffer around the residents) among study participants of the Swedish Mammography Cohort (SMC) residing in Uppsala County, Sweden, grouped by urbanization type (based on degree of urbanization by the demographic statistical areas [DeSO]).

Generally, we only found small differences in annual average exposure level across the categories of our selected sociodemographic (Figure 2 and Figures S4–10; http://links.lww.com/EE/A250) and lifestyle variables (Figures S11–14; http://links.lww.com/EE/A250). However, urbanization type appeared clearly related to the level of exposure, with urban residency consistently associated with higher levels of long-term air pollution and road traffic noise, but to lower levels of greenness in comparison to suburban and rural residency (Figure 2).

**Figure 3.**
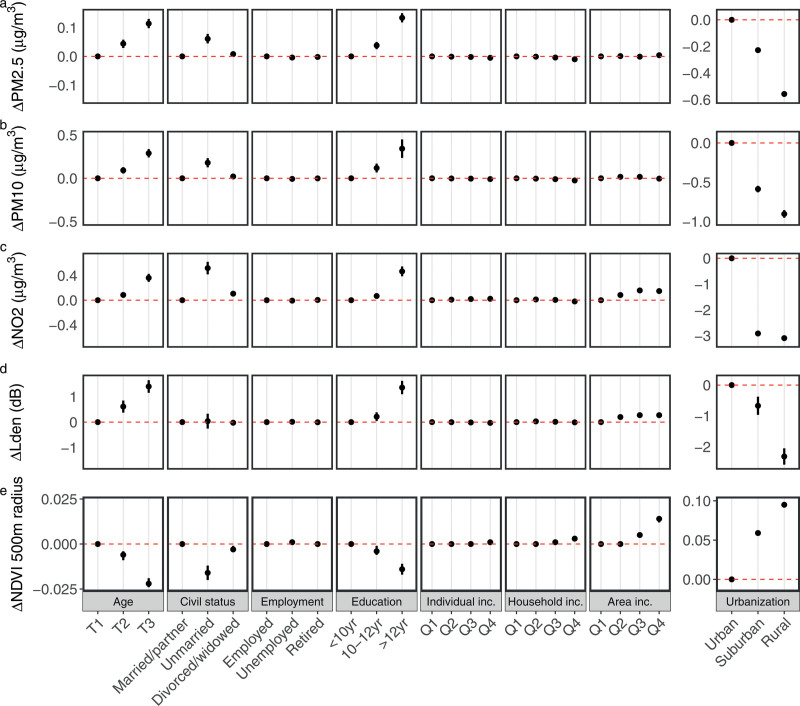
Associations between sociodemographic determinants and environmental exposures. GEE-derived beta-coefficients (β) and 95% confidence intervals (CI) of associations between various socioeconomic determinants and exposure to air pollution (A) PM_2.5_, (B) PM_10_, (C) NO_2_, (D) road traffic noise (*L*_den_), and (E) greenness (NDVI within a buffer of 500 m radius) at the residence adjusted for calendar years. Each row represents coefficients of one model where the predictors are the sociodemographic factors on the *x*-axes and the response variables are the environmental exposures on the *y*-axes.

The results from the GEE analyses (Figure 3 and corresponding numbers in Table S1; http://links.lww.com/EE/A250) confirmed that the most important predictor of high exposure to air pollution and road traffic noise and to low-level greenness was urbanization type. The exposure to PM_2.5_, PM_10_, NO_2_, and road traffic noise was markedly lower in suburban and rural areas, as compared with urban areas. For example, β for NO_2_ was −2.92 (95% CI = −3.00, −2.83) and −3.10 (95% CI = −3.18, −3.01) µg/m^3^ in suburban and rural areas, respectively, in comparison to urban areas. For greenness, the opposite held true with β of 0.059 (95% CI = 0.056, 0.062) and 0.095 (95% CI = 0.092, 0.098) for suburban and rural areas, respectively, in comparison to urban areas.

There were also clear patterns of higher exposure to particles, nitrogen dioxide and road traffic noise, and lower levels of greenness, among the elderly and among women with a higher level of education. For instance, the β for road traffic noise was 1.40 (95% CI = 1.15, 1.65) dB *L*_den_ among the oldest women (born 1914–31) in comparison to the youngest (born 1941–48). Furthermore, β for PM_10_ was 0.34 (95% CI = 0.24, 0.45) µg/m^3^ among women with postsecondary/postgraduate education compared with those with presecondary education up to 9 years only. Civil status also appeared to be a predictor of some importance, indicating that unmarried women tended to have higher exposure to particles and nitrogen dioxide and lower levels of greenness in comparison to women who were married or living with a partner and to those divorced or widowed. For area-based income, we observed a positive association with greenness, for example, the most affluent group had statistically significant higher levels of NDVI close to their residence (β 0.014, 95% CI = 0.012, 0.015) in comparison to the least affluent group. Less clear or no associations were indicated for employment status, individual income, and household income.

Adding the selected individual lifestyle variables, that is, alcohol, smoking, exercise, and BMI, to the regression model as additional explanatory factors of urban environmental exposure did not significantly alter our conclusions from the main model (Figure S15; http://links.lww.com/EE/A250). Despite a slight tendency of a higher particle and nitrogen dioxide exposure and a lower greenness exposure among former and current smokers in comparison to never smokers, no pronounced associations were found between lifestyle and environmental exposure.

When stratifying the population on urbanization type, similar results as in the total population were found in the urban subpopulation, however, somewhat different, or less pronounced, patterns of association emerged in the suburban and rural subpopulations (Figures S16–18; http://links.lww.com/EE/A250). For instance, the associations with age, education, and civil status were clearly diluted and approached unity. In some instances, for example, for road traffic noise and age, the associations were even reversed in the suburban subpopulations compared with the total population. There were also indications of nonlinear associations between area-based income and air pollution and greenness in the suburban subpopulation. Furthermore, NO_2_ was clearly positively associated with area-based income in the rural subpopulation, indicating higher exposure in the higher income groups.

## Discussion

In this 20-year follow-up study of women from Uppsala County, Sweden, we found an overall declining trend in the exposure to air pollution over time which is consistent with the overall trend in Uppsala County for the corresponding time period.^23^ However, the exposure to road traffic noise and greenness remained constant. Of the independent variables under study, urbanization type was the single most important predictor of exposure, with higher levels of air pollution and road traffic noise and lower levels of greenness among women living in urban areas as compared with those living in suburban and rural areas. Age and education were also positively associated with both air pollution and noise, but inversely associated to greenness. Furthermore, civil status and area-based income also proved to be of some importance for individual environmental exposure. No pronounced associations were found between lifestyle and environmental exposures, however, stratification by urbanization type indicated strongest associations in the urban subpopulation.

Increasing evidence indicates that exposure to urban environmental factors such as air pollution and traffic noise may be harmful to human health.^1–6^ Greenness, on the other hand, may help to improve population health.^8^ Environmental health inequalities, implying that some groups have a greater share of environmental pollution than others, have been detected both within and between the European population.^10,11^ Deprived populations may suffer from a “double burden,” experiencing not only increased exposure but also increased vulnerability to the effects of exposure, thus leading to more pronounced health effects within these groups. However, the evidence in this area is still inconclusive. By its focus on sociodemographic and lifestyle variables as determinants of individual-level environmental exposure, the present study adds valuable scientific evidence in this area. Based on our findings, we cannot confirm the hypothesis of a higher environmental burden solely in socioeconomic deprived and vulnerable groups. Rather, our results point to a more mixed pattern with higher exposure levels both among groups considered vulnerable, for example, the elderly, and in nonvulnerable groups, for example, well-educated women.

In a global review of air pollution and socioeconomic disparities by Hajat et al,^24^ it was concluded that while most North American studies, and research from Asia and Africa, showed that communities with low SES experience higher concentrations of air pollutants, findings from European studies appear more mixed. The present study of women from Uppsala County, Sweden, adds to that picture, pointing to a rather complex interplay between environmental exposure, individual and area-based SES, and degree of urbanization. Similar to conclusions drawn by Hajat et al, we generally only found small absolute differences in pollutant exposure across the categories of the sociodemographic determinants under study. Like Hajat et al, we also observed exceptions to the overall patterns, in particular when stratifying on urbanization type where some sociodemographic factors showed diluted or even opposite associations with the environmental exposures in the suburban and rural subpopulation compared with the total population and urban subpopulation. This may reflect differences in the city building and varying clustering of groups with different sociodemographic compositions within urban, suburban, and rural areas, respectively.

Relatively few studies have assessed the distribution of noise exposure across different sociodemographic groups and the evidence is therefore still inconclusive. A recent review by Dreger et al^25^ of eight studies performed in France, Germany, and the United Kingdom found mixed results on how environmental noise exposure was linked to SES characteristics. Studies using only one or a low number of SES indicators pointed to a disadvantage of people with low SES regarding noise exposure. However, when summarizing the results of all the included studies, opposing results were found for single SES variables both within and across studies. For example, low individual level of education has in some instances been associated with higher noise exposure, but not consistently throughout the analyses of individual datasets and across studies.^26,27^ Findings from the present study add to this ambivalent picture, indicating a positive association between road traffic noise and education in the urban subpopulation only. Moreover, the results by Dreger et al^25^ suggest a lower noise exposure in older people which in our study only held true in the suburban subpopulation.

Most of the studies investigating greenness exposure in different groups of society use ecological design, but some reports are based on individual-level data. Overall, there seems to be a difference considering the results from ecological and individual-level studies where the ecological studies consistently show that deprived areas have lower greenness availability than more affluent ones, whereas the associations in individual-level studies are rather mixed.^28^ A previous study on neighborhood socioeconomic factors and green structure in urban and suburban municipalities in Stockholm County indicated that the direction of the associations, for example, between greenness and area-based mean income, differed according to municipality type.^16^ These results were not confirmed by the present investigation which indicated higher levels of greenness among the most affluent groups in all areas, urban, suburban, and rural, although the association was nonlinear in the suburban subpopulation.

In this study, we included five individual-, one household, and two small area-level variables, based on previous literature, data availability, quality of variables, and relevance to this cohort and area. This covers more of the socio-demography than commonly used in most exposure-health-association studies (e.g., see,^29–31^ etc.). However, some variables which may be of general interest were not included. For example, we did not include ethnicity since the SMC was very uniform in that regard. Overall, it is difficult to make general statements about the nature of environmental inequalities. This may stem from methodological differences between the studies, for instance in assessment and definition of exposure, differences relating to the sociodemographic indicators used. However, it may also point to natural variations within the society relating to urban form and population composition. Based on the findings from the present study, associations between environmental exposure and sociodemographic characteristics appear more pronounced in densely populated areas. In particular, it seems that women with higher education tend to live in the more polluted city center, possibly due to the attractiveness of the area and higher rental and housing prices. Generally, however, this cohort study of women from Uppsala County, Sweden, adds to the evidence of a mixed pattern of association between environmental exposure and sociodemographic characteristics and highlights the need for population and location-specific knowledge of the occurrence and distribution of environmental pollution within the population as a basis for public health prevention and sustainable urban planning.

A limitation of the present study is the restricted generalizability. The SMC consists of elderly women, mainly Swedish-born, living in a relatively low environmental polluted and high greenness exposed study area. Thus, the results may not be entirely valid for men, for populations with a more varied ethnical background, or for regions with different ranges of exposure. Additionally, the city of Uppsala, the largest agglomeration in the County, is a university city with few industries which may also limit the generalizability, for example, in terms of education and employment status. Still, however, the results do cast some light into important sociodemographic differences in environmental exposure within the Swedish population which is of relevance for populations in other regions and countries. Another limitation of the study may be the potential occurrence of spatial misalignment, mainly driven by the introduction of the two area-level variables (mean income and urbanization type), which may have led to less reliable standard errors on the regression coefficient regulating the strength of association. However, we believe that this is not a major point of concern of our study since we use similar spatial and time scales for all exposures, and individual-level information on most sociodemographic variables. Furthermore, to minimize spatial correlation, we assigned the contextual variables to each study participant on the smallest spatial scale available (i.e., DeSO). Loss to follow-up could pose a problem in long-term cohort studies, however, we do not believe it to be a major problem here since we were able to follow all but 522 women through linkage to registers. Furthermore, the average exposure among women lost to follow-up (e.g., due to moving out of the study area) and those remaining in the study was only marginal which implies that the loss to follow-up was not driven by a higher environmental burden. However, we still did observe a slight shift in the population composition over time, indicating that (nonsmoking) women with higher SES live longer. Since older women also tended to be more exposed, this may have led to an overestimation of the association.

This study has several strengths. Firstly, its longitudinal design enabled investigations of environmental exposure inequalities over time, which to our knowledge has not been done previously. To achieve this, we combined longitudinal exposure information with time-varying register information on sociodemographic variables and, additionally, updated questionnaire-derived information on a set of key lifestyle variables. Secondly, we assessed not only one but a multitude of sociodemographic determinants simultaneously in the models and were thus able to assess different aspects of environmental inequality. This is of crucial importance to achieve a more complete picture of the complexity of the area. Thirdly, we used individual data on both the exposures and the sociodemographic variables. The use of smaller levels of geography, preferably individual-level information, in these types of studies is advocated to improve the reliability and accuracy of the study^.32,33^ Finally, by providing results stratified on urbanization type, we were able to detect potentially important differences in the patterns of associations depending on degree of urbanization which may otherwise have been masked.

In conclusion, this time-varying study confirms the picture of a complex interplay in Europe between environmental exposure, individual and area-based sociodemographic characteristics, and degree of urbanization. Urban area residency, high age, high education and, partly, being unmarried, were found to be associated with higher exposure to particles, nitrogen dioxide, and road traffic noise, and to a lower greenness. However, less clear patterns emerged for other determinants (employment status, individual and household income), lifestyle variables, and in strata of suburban and rural areas. The study thus highlights the need for population and location-specific knowledge as a basis for public health prevention and sustainable urban planning.

## ACKNOWLEDGMENTS

The authors wish to thank the participants of the SMC and the Swedish Infrastructure for Medical Population-Based Life-Course and Environmental Research (SIMPLER) with support from the Scientific Research Council (2017-00644) for providing valuable data. The computations were performed on resources provided by SNIC-SENS through the Uppsala Multidisciplinary Center for Advanced Computational Science (UPPMAX) under Project SIMP2020009. We also wish to thank Kristina Eneroth, SLB Analys, for providing expert information on air pollution dispersion modeling, Östen Axelsson for statistical advice, Emilie Helte sharing her knowledge of the questionnaire data from SMC and Åsa Persson for guidance of the NDVI exposure assessment.

## Supplementary Material
