# Supplementary material for: Sociodemographic inequalities in long-term exposure to air pollution, road traffic noise, and greenness: A population-based cohort study of women
Source: Environ Epidemiol. 2023 Dec 1;7(6):e279. doi: 10.1097/EE9.0000000000000279 (PMC11189682; doi:10.1097/EE9.0000000000000279)
Supplement: Supplementary file 1 [file ee9-7-e279-s001.docx]

**Supplementary Material**

**Sociodemographic inequalities in long-term exposure to air pollution, road traffic noise, and greenness – A population-based cohort study of women**

Lara Stucki^a,c^, Staffan Betnér^a,b^, Jenny Selander^a^, Mare Lõhmus^a,b^, Agneta Åkesson^a^, Charlotta Eriksson^a,b^

^a^ Institute of Environmental Medicine, Karolinska Institutet, Nobels väg 13, 171 77 Stockholm, Sweden

^b^ Centre for Occupational and Environmental Medicine, Region Stockholm, Solnavägen 4, 113 65 Stockholm, Sweden

^c^ Corresponding author: Institute of Environmental Medicine, Karolinska Institutet, Nobels väg 13, 171 77 Stockholm, Sweden, [Lara.Stucki@ki.se](mailto:Lara.Stucki@ki.se)

**Table of Content**

**Figures**

**Figure S1.** **Flow-chart of the Swedish Mamography Cohort (SMC).**

**Figure S2**. **Pearson correlation coefficients among sociodemographic determinants.**

**Figure S3. Histograms of environmental exposure.**

**Figure S4. Time-trends of environmental exposures by tertiles of age**.

**Figure S5. Time-trends of environmental exposures by civil status.**

**Figure S6. Time-trends of environmental exposures by employment status.**

**Figure S7. Time-trends of environmental exposures by levels of education.**

**Figure S8. Time-trends of environmental exposures by quartiles of individual income.**

**Figure S9. Time-trends of environmental exposures by quartiles of household income.**

**Figure S10.** **Time-trends of environmental exposures by quartiles of area-based income.**

**Figure S11.** **Time-trends of environmental exposures by tertiles of alcohol consumption.**

**Figure S12. Time-trends of environmental exposures by smoking status.**

**Figure S13.** **Time-trends of environmental exposures by BMI.**

**Figure S14. Time-trends of environmental exposures by categories of exercising**

**Figure S15. Sensitivity analysis: Associations between sociodemographic determinants and environmental exposures further adjusted for lifestyle variables.**

**Figure S16. Stratification: Associations between sociodemographic determinants and environmental exposures restricted to the urban area**.

**Figure S17. Stratification: Associations between sociodemographic determinants and environmental exposures restricted to the suburban area**.

**Figure S18. Stratification: Associations between sociodemographic determinants and environmental exposures restricted to the rural area**.

**Table**

**Table S1. Associations between sociodemographic determinants and environmental exposures**.

**
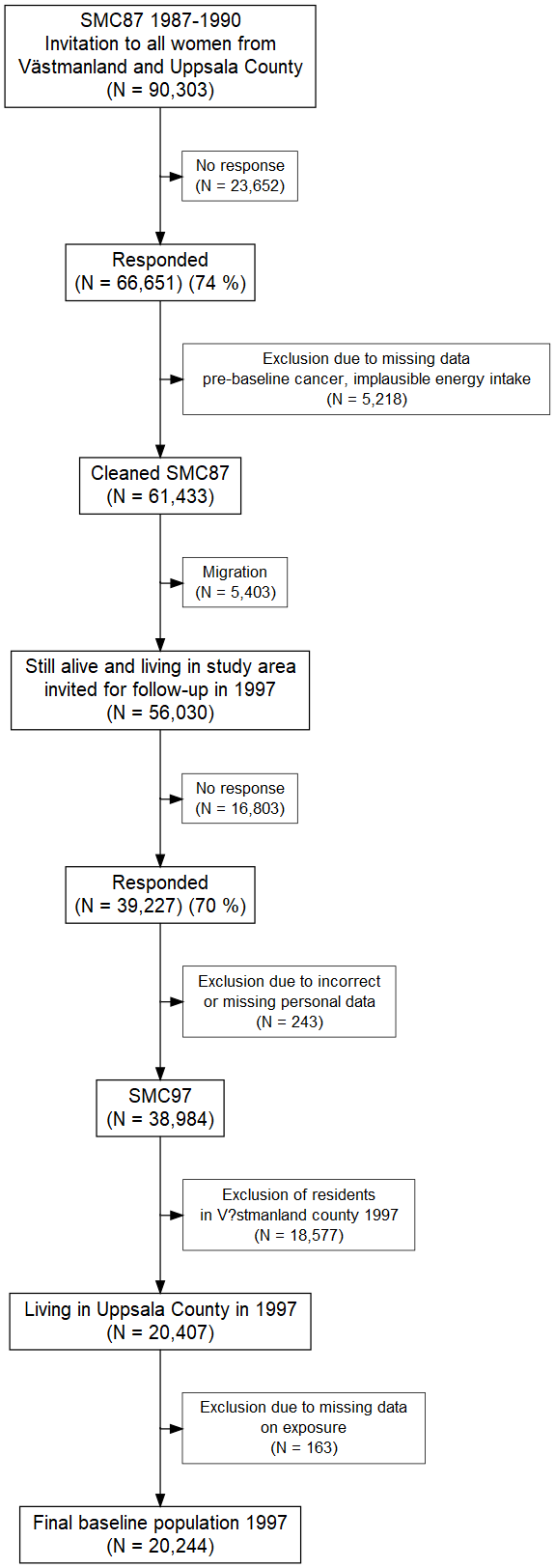
**

**Figure S1.** **Flow-chart** **of the Swedish Mamography Cohort (SMC).**

**
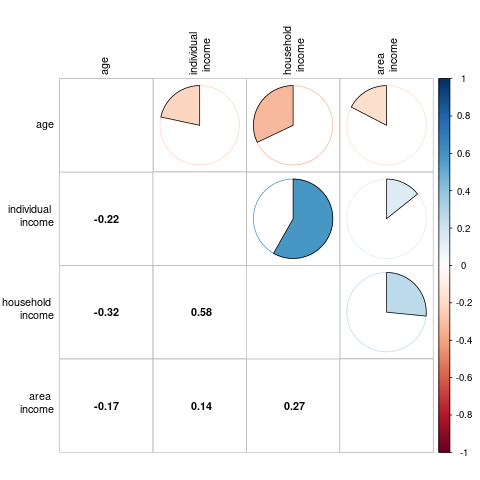
**

**Figure S2. Pearson correlation coefficients among sociodemographic determinants:** Pair-wise Pearson correlation coefficients among the continuous socioeconomic variables, individual, household, and area-based income at baseline (1997).

**
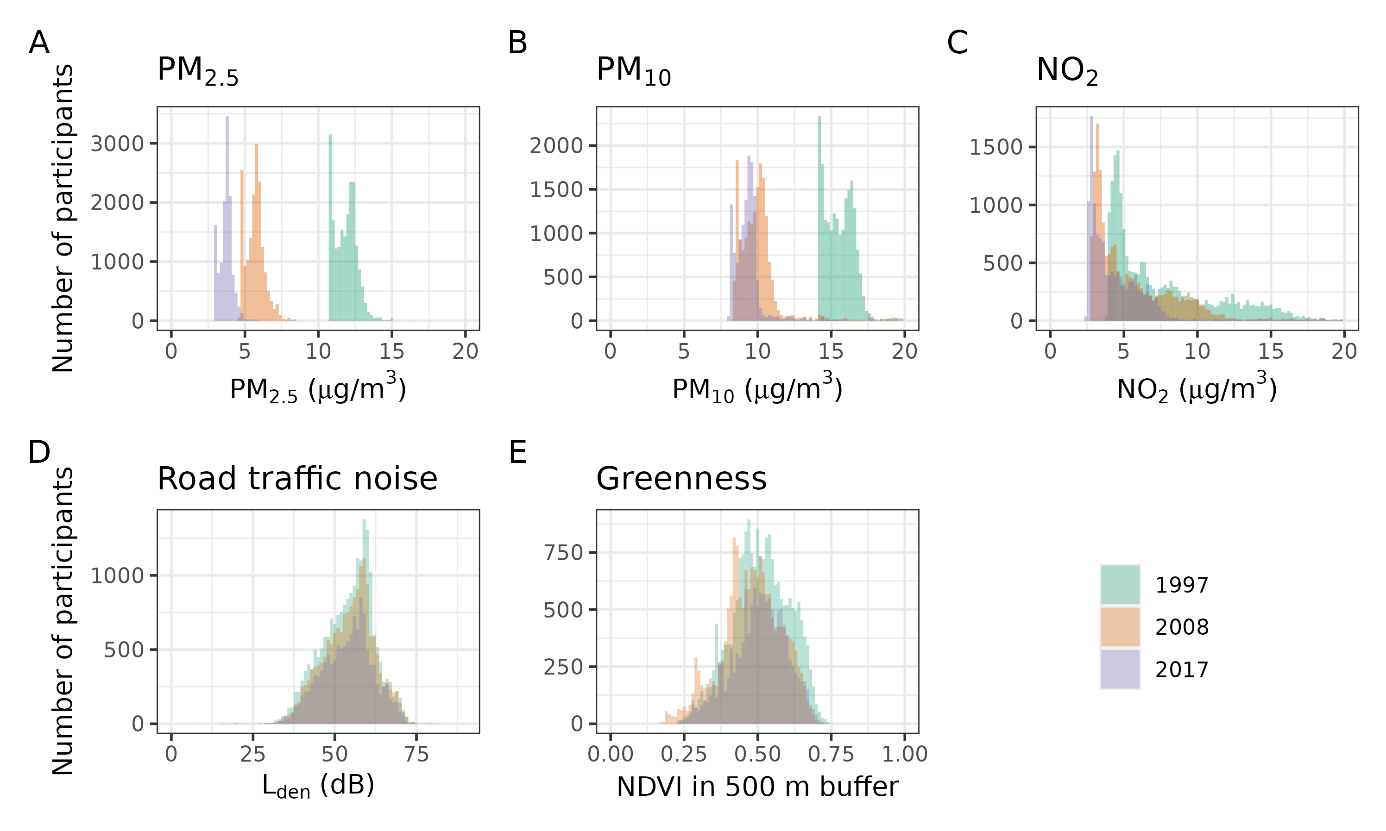
**

**Figure S3. Histograms of environmental exposure:** Frequency histograms of exposure to air pollution (PM_2.5_, PM_10_, NO_2_), road traffic noise (L_den_) and greenness (NDVI in 500 m buffer) in 1997, 2008 and 2017 among study participants of the Swedish Mammography Cohort residing in Uppsala County, Sweden.

**Note:** **PM_10_:** In 1997 522 (2.5 %) observations/participants were on average exposued to >20 µg/m^3^. In 2008 17 (0.10 %) observations/participants were on average exposued to >20 µg/m^3^.In 2017 5 (0.040 %) observations/participants were on average exposued to >20 µg/m^3^. **NO_2_:** In 1997 741 (3.7 %) observations/participants were on average exposued to >20 µg/m^3^. In 2008 502 (2.9 %) observations/participants were on average exposued to >20 µg/m^3^. In 2017 180 (1.4 %) observations/participants were on average exposued to >20 µg/m^3^. **Ranges** of enviromental exposures are for PM_2.5_ 2.72-17.02 µg/m^3^, PM_10_ 7.33-52.43 µg/m^3^, NO_2_ 1.35-50.93 µg/m^3^, L_den_ 5.20-83.50 dB, and NDVI within a 500 m buffer 0.169-0.813 including all observations throughout the study period.


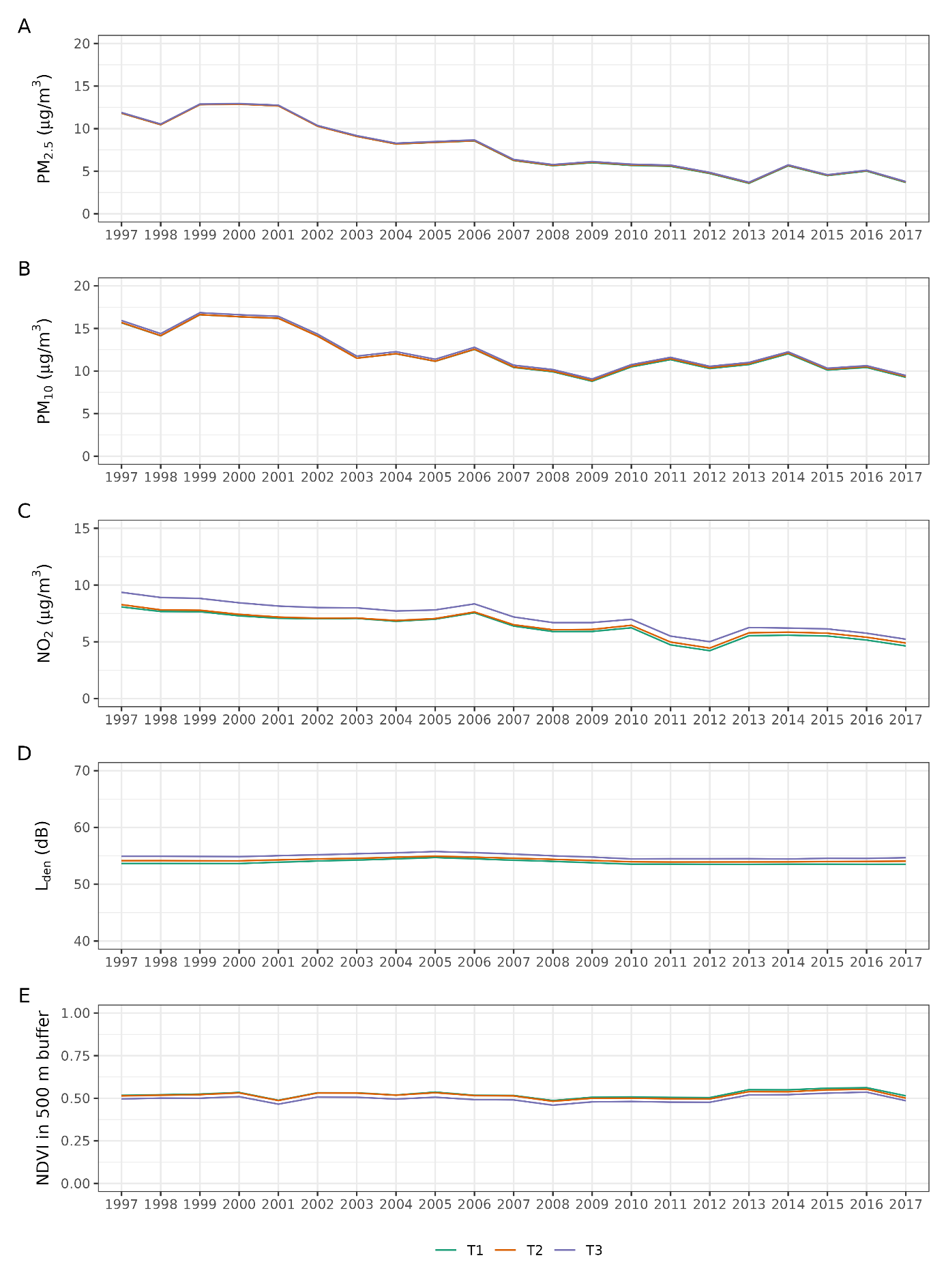


**Figure S4.** **Time-trends of environmental exposures by tertiles of age:** Time-trends of the environmental exposure to A) PM_2,5_, B) PM_10_, C) NO_2_, D) road traffic noise and E) greenness (500 m radius buffer around the residents) among study participants of the Swedish Mammography Cohort (SMC) residing in Uppsala County, Sweden, grouped by tertiles of age.


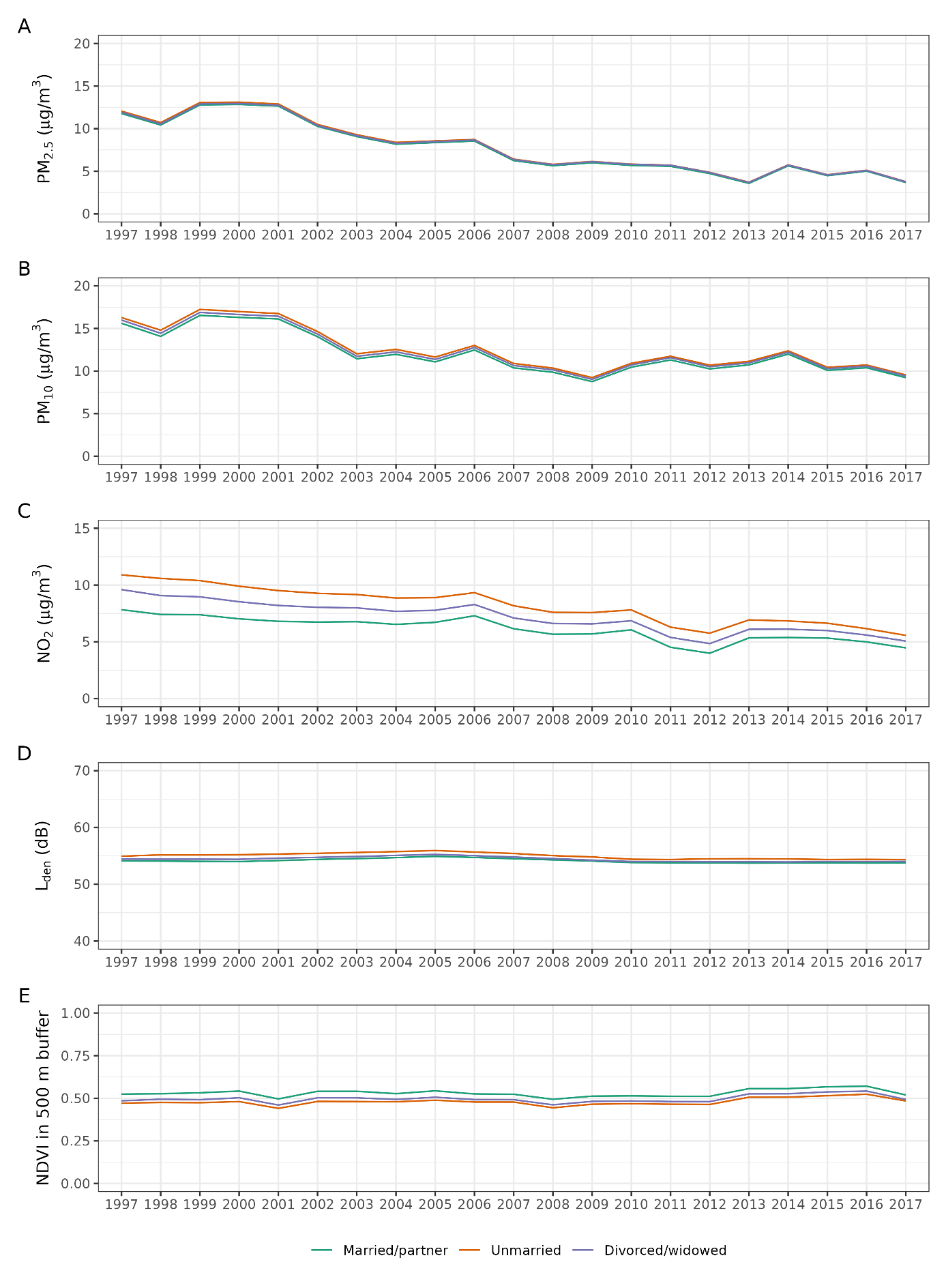


**Figure S5. Time-trends of environmental exposures by civil status:** Time-trends of the exposure to A) PM_2,5_, B) PM_10_, C) NO_2_, D) road traffic noise and E) greenness (500 m radius buffer around the residents) among study participants of the Swedish Mammography Cohort (SMC) residing in Uppsala County, Sweden, grouped by civil status.


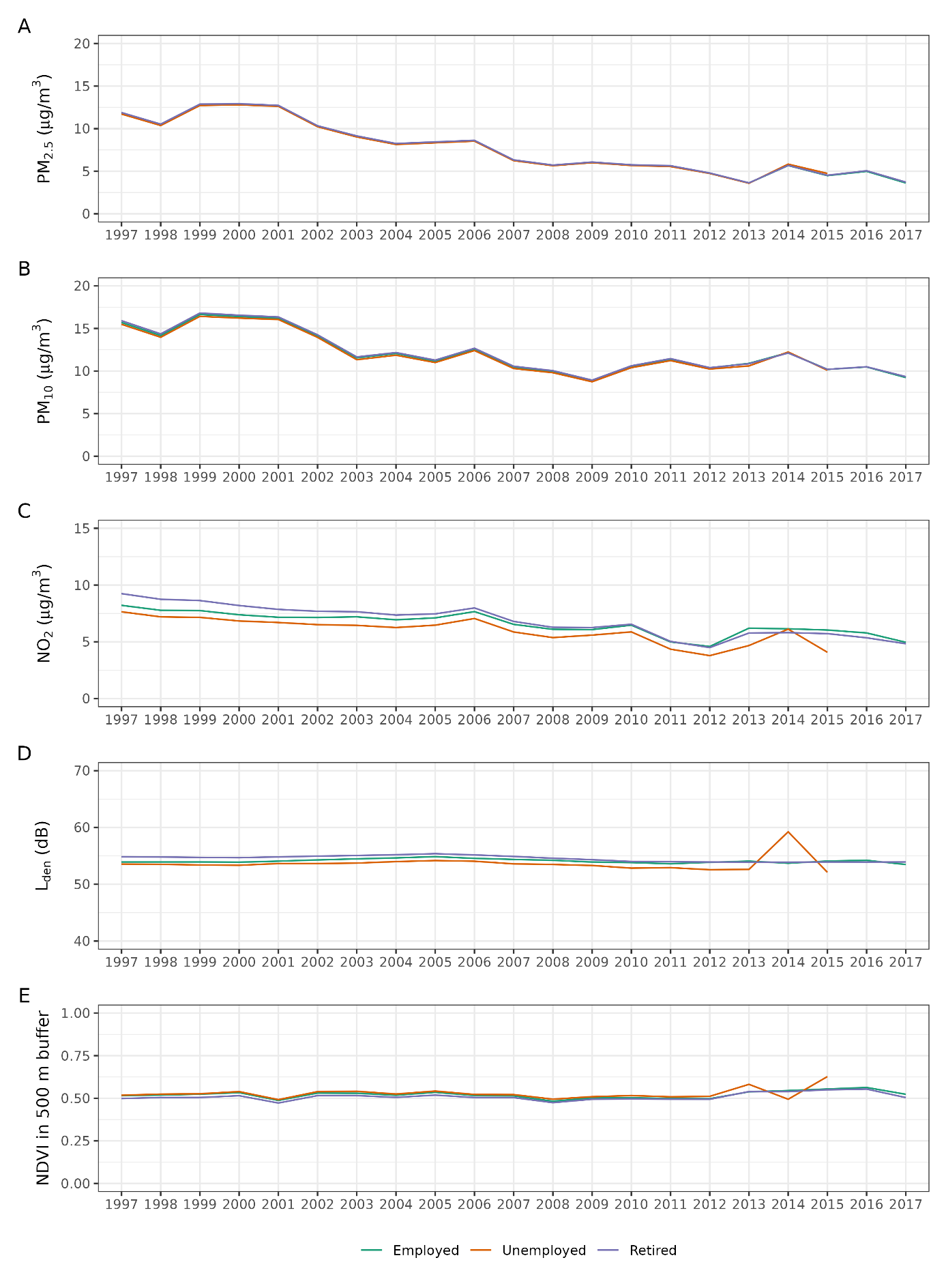
 **Figure S6. Time-trends of environmental exposures by employment status:** Time-trends of the exposure to A) PM_2,5_, B) PM_10_, C) NO_2_, D) road traffic noise and E) greenness (500 m radius buffer around the residents) among study participants of the Swedish Mammography Cohort (SMC) residing in Uppsala County, Sweden, grouped by employment status.


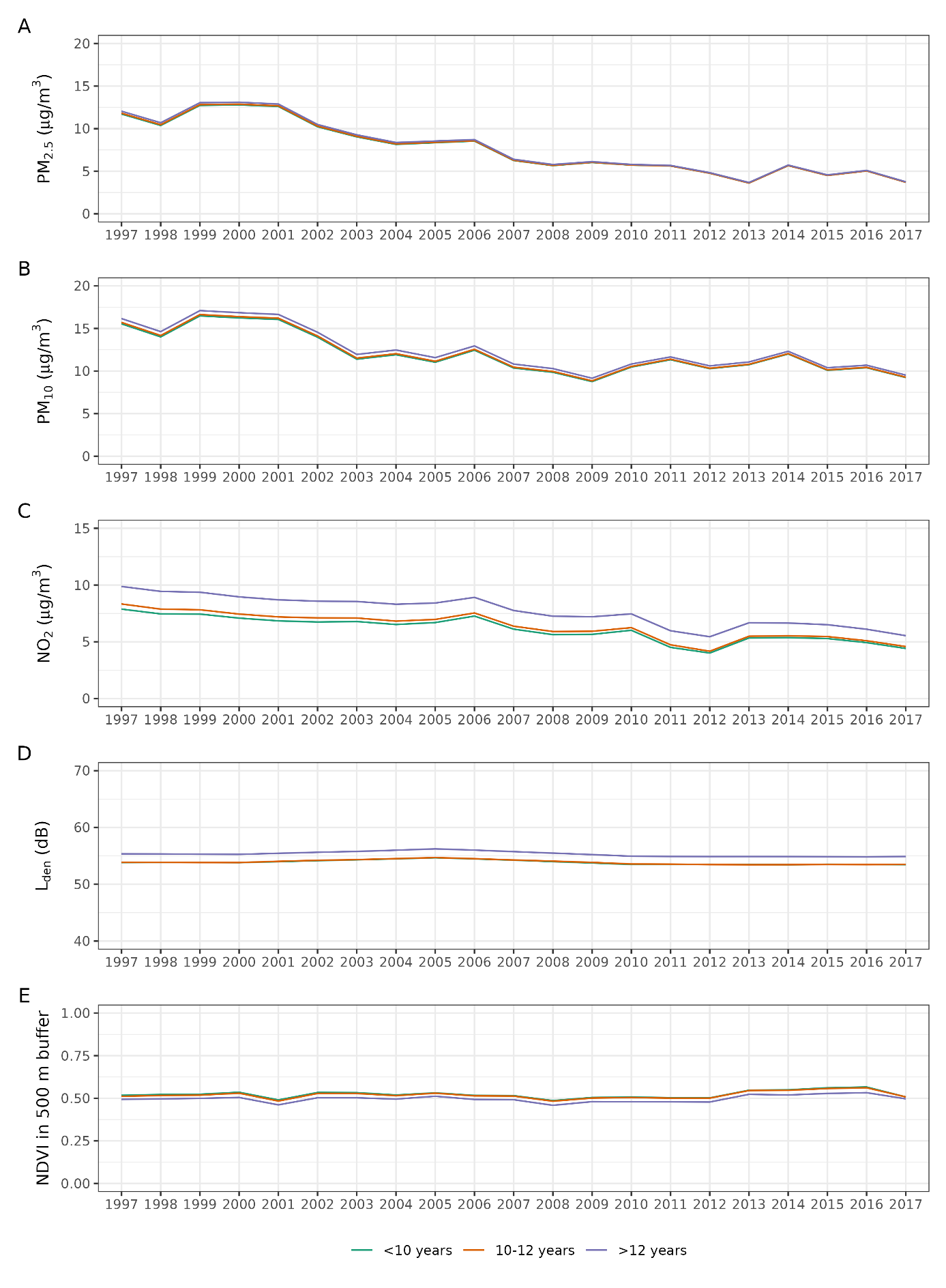


**Figure S7. Time-trends of environmental exposures by levels of education:** Time-trends of the exposure to A) PM_2,5_, B) PM_10_, C) NO_2_, D) road traffic noise and E) greenness (500 m radius buffer around the residents) among study participants of the Swedish Mammography Cohort (SMC) residing in Uppsala County, Sweden, grouped by highest achieved educational level.

**
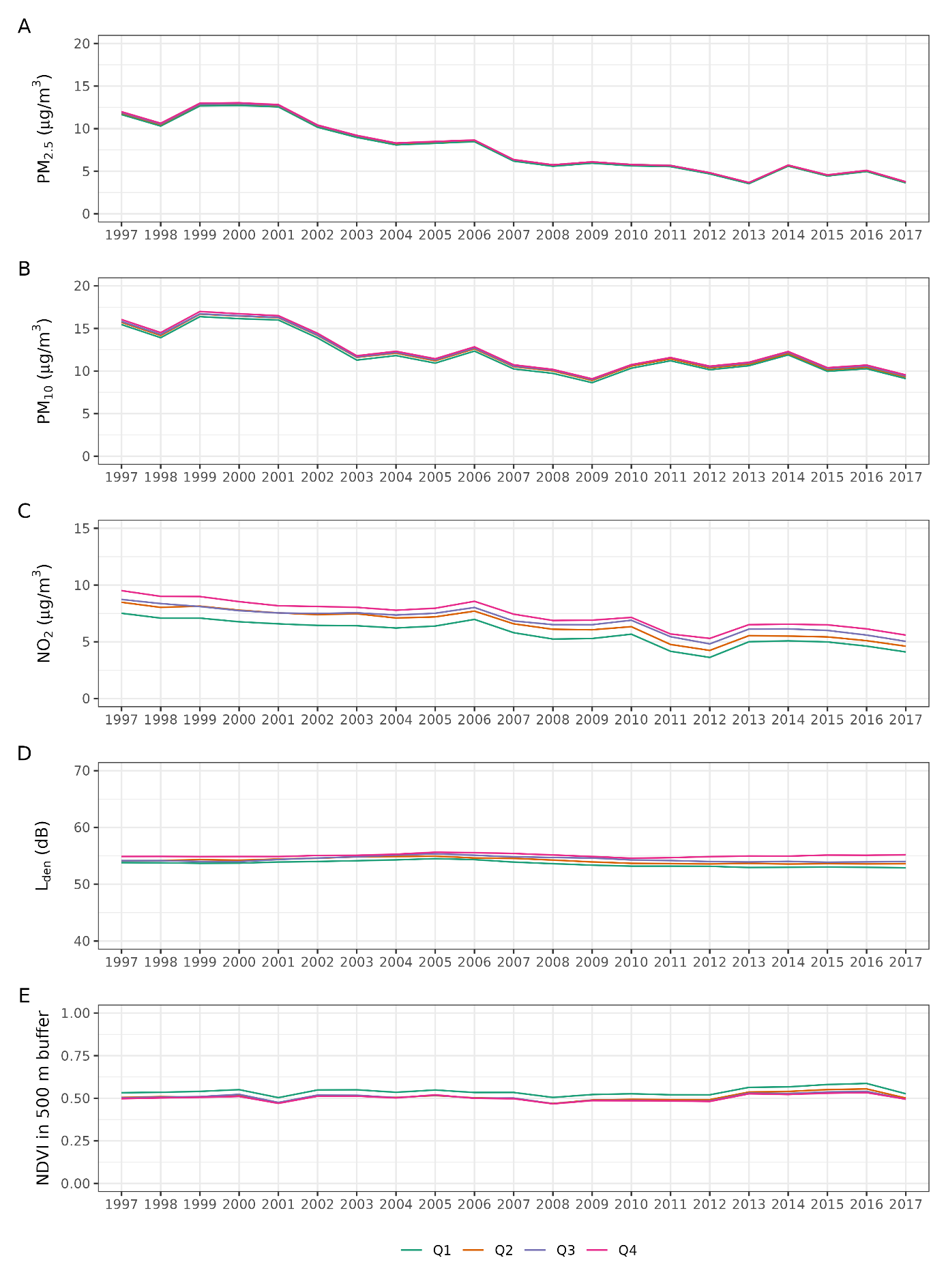
**

**Figure S8. Time-trends of environmental exposures by quartiles of individual income:** Time-trends of the exposure to A) PM_2,5_, B) PM_10_, C) NO_2_, D) road traffic noise and E) greenness (500 m radius buffer around the residents) among study participants of the Swedish Mammography Cohort (SMC) residing in Uppsala County, Sweden, grouped by quartiles of individual income.


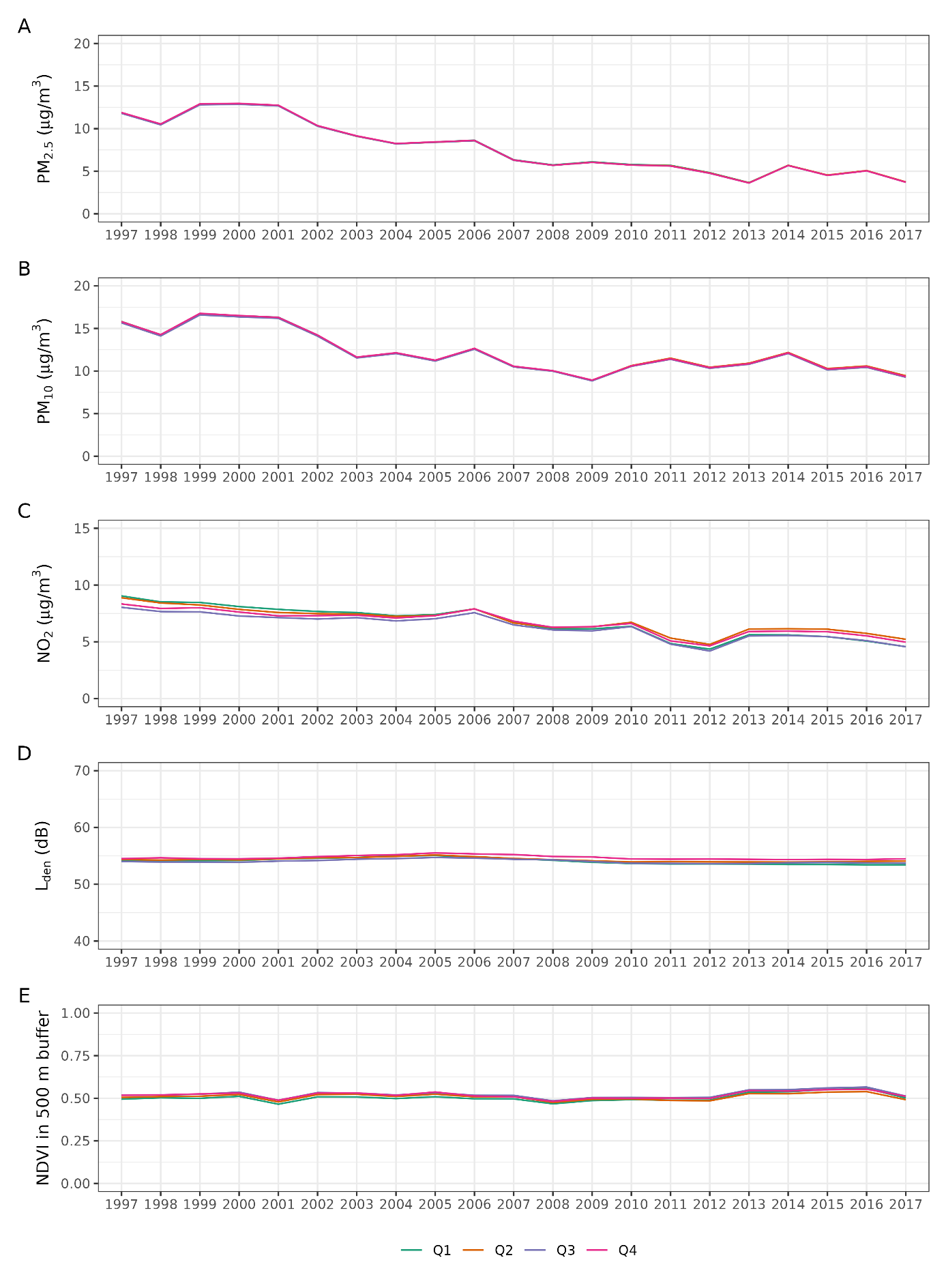


**Figure S9. Time-trends of environmental exposures by quartiles of household income:** Time-trends of the exposure to A) PM_2,5_, B) PM_10_, C) NO_2_, D) road traffic noise and E) greenness (500 m radius buffer around the residents) among study participants of the Swedish Mammography Cohort (SMC) residing in Uppsala County, Sweden, grouped by quartiles of household income.


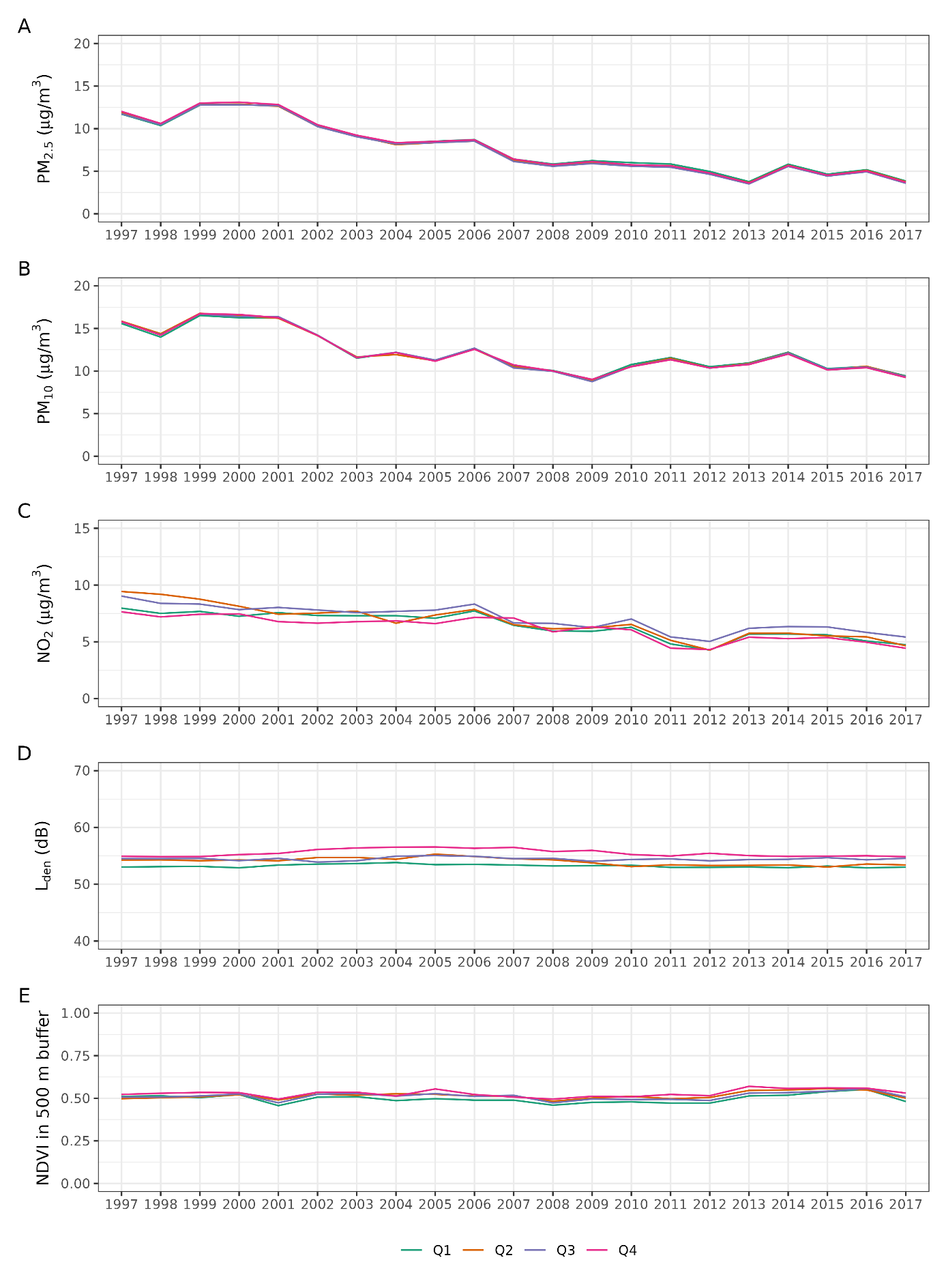


**Figure S10.** **Time-trends of environmental exposures by quartiles of area-based income:** Time-trends of the exposure to A) PM_2,5_, B) PM_10_, C) NO_2_, D) road traffic noise and E) greenness (500 m radius buffer around the residents) among study participants of the Swedish Mammography Cohort (SMC) residing in Uppsala County, Sweden, grouped by quartiles of area-based income based on DeSO areas.


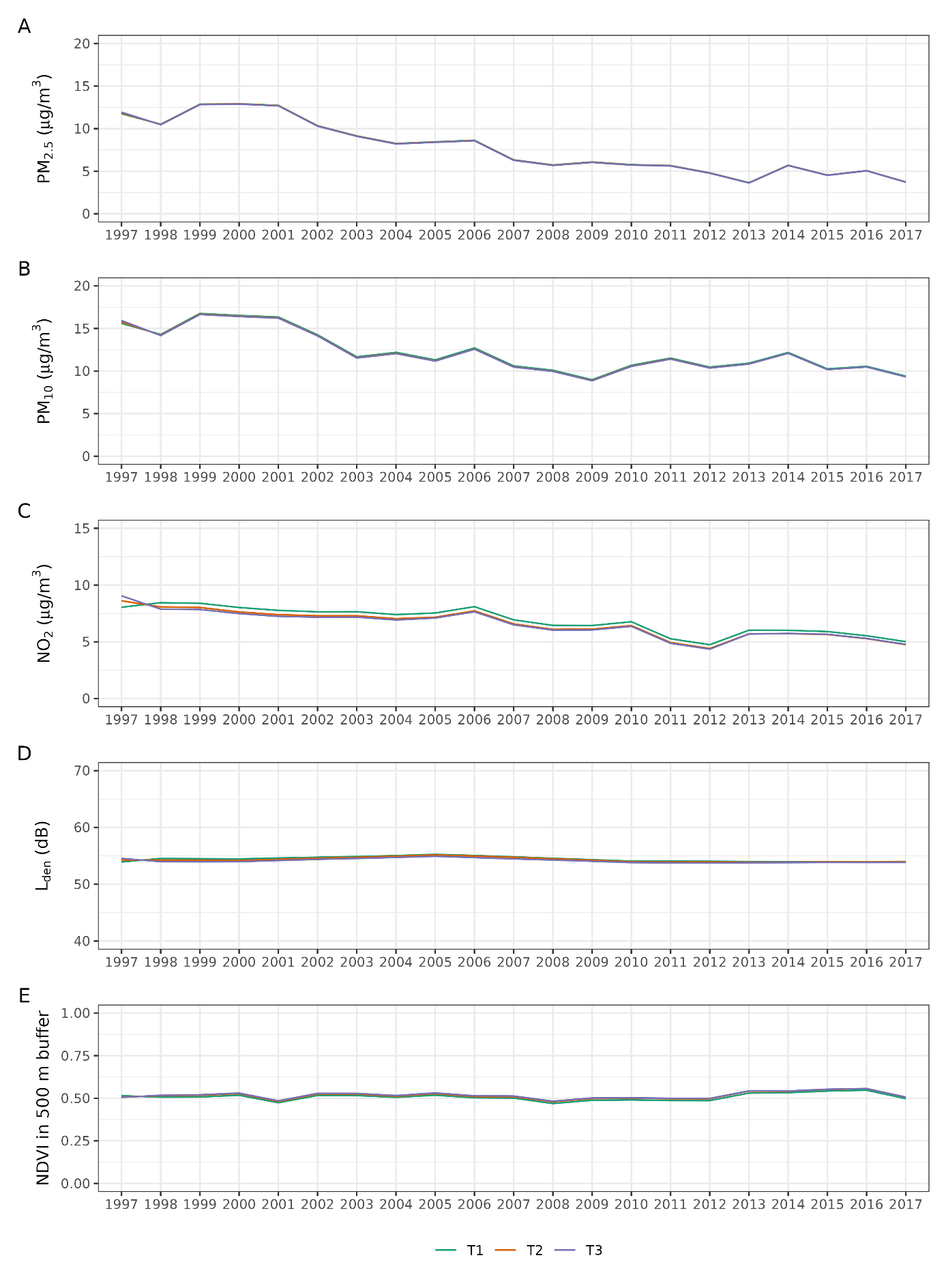


**Figure S11. Time-trends of environmental exposures by tertiles of alcohol consumption:** Time-trends of the exposure to A) PM_2,5_, B) PM_10_, C) NO_2_, D) road traffic noise and E) greenness (500 m radius buffer around the residents) among study participants of the Swedish Mammography Cohort (SMC) residing in Uppsala County, Sweden, grouped by tertiles of alcohol consumption.


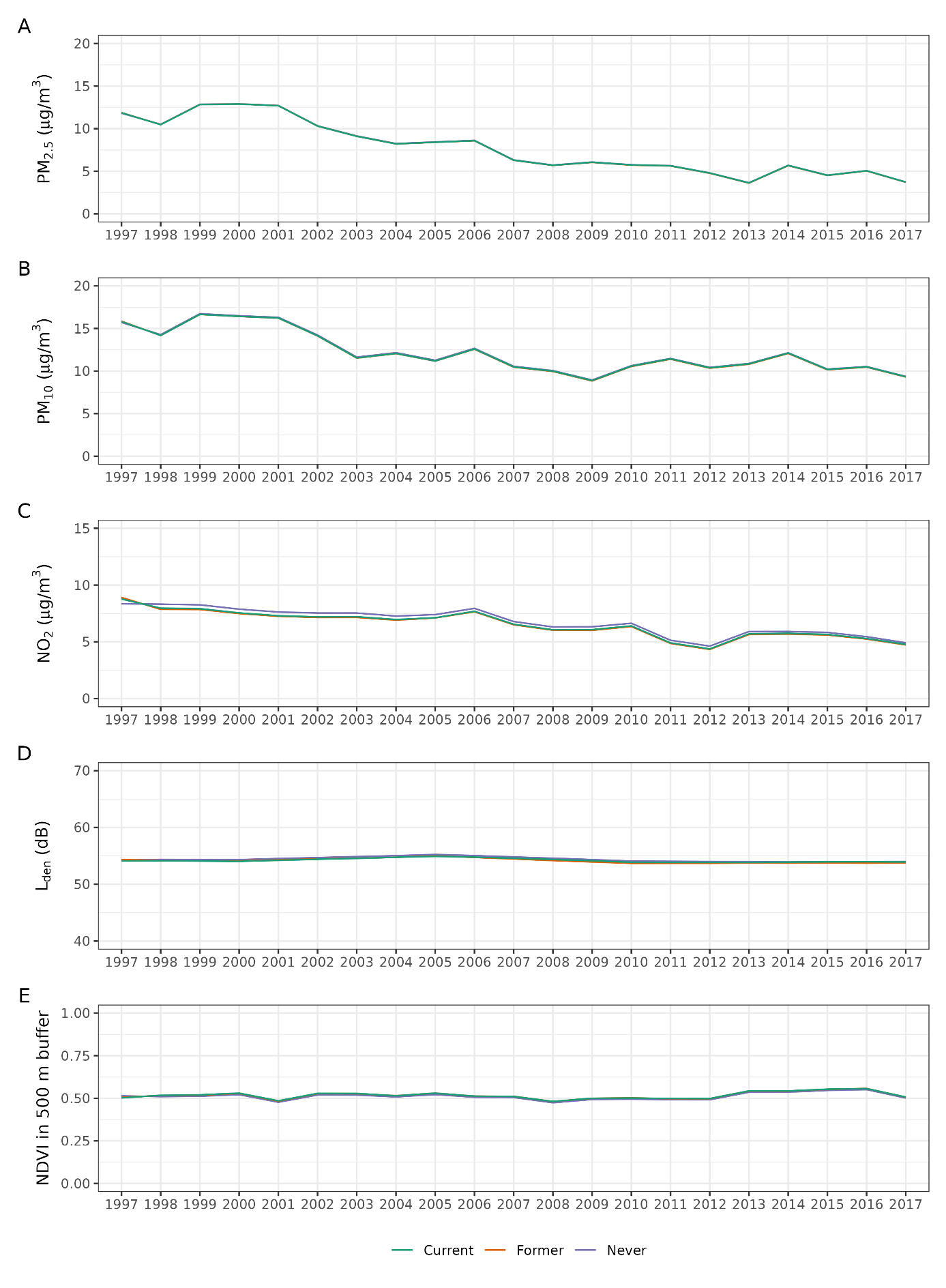


**Figure S12.** **Time-trends of environmental exposures by smoking status:** Time-trends of the exposure to A) PM_2,5_, B) PM_10_, C) NO_2_, D) road traffic noise and E) greenness (500 m radius buffer around the residents) among study participants of the Swedish Mammography Cohort (SMC) residing in Uppsala County, Sweden, grouped by smoking status.


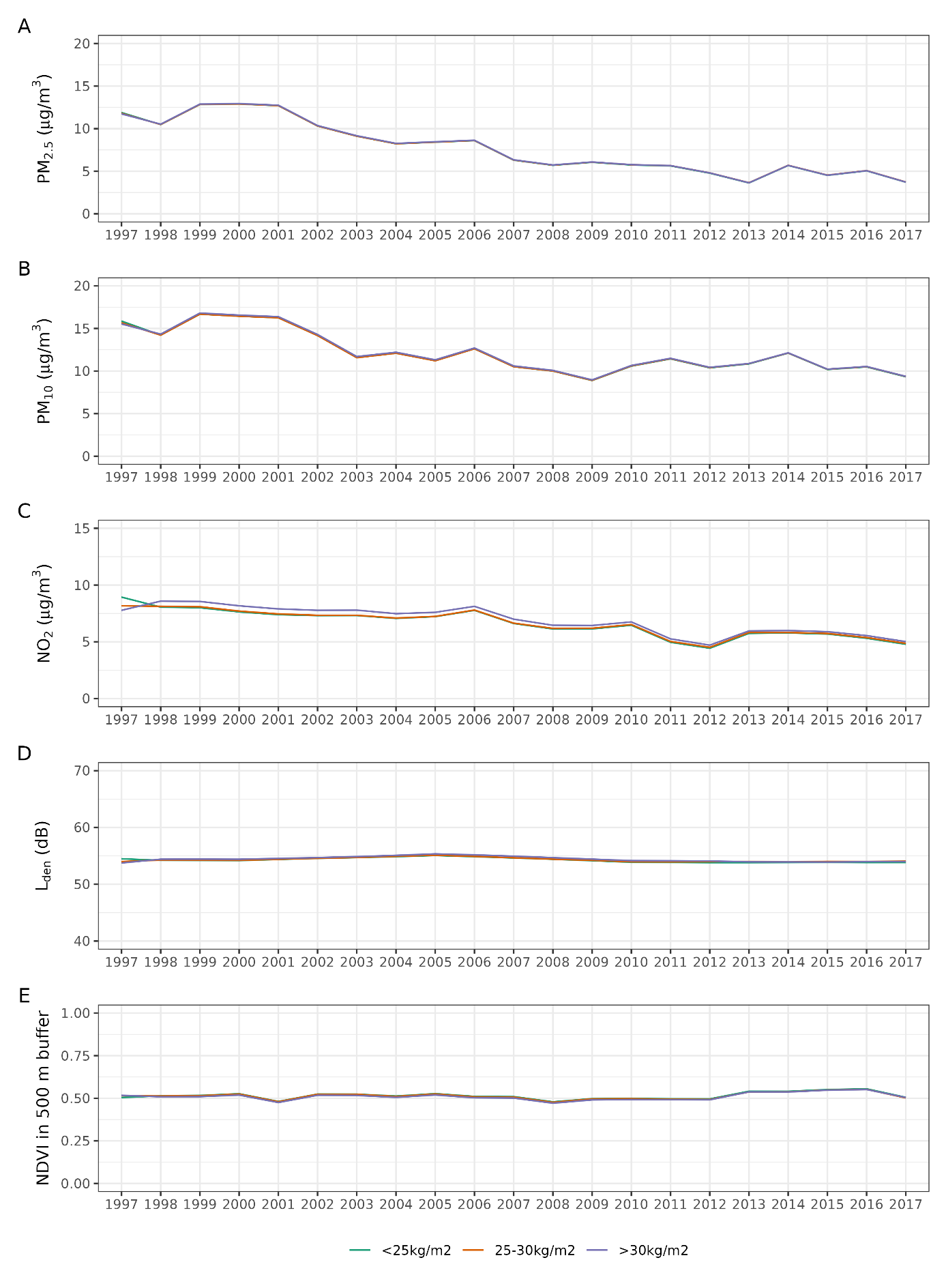


**Figure S13.** **Time-trends of environmental exposures by categories of BMI:** Time-trends of the exposure to A) PM_2,5_, B) PM_10_, C) NO_2_, D) road traffic noise and E) greenness (500 m radius buffer around the residents) among study participants of the Swedish Mammography Cohort (SMC) residing in Uppsala County, Sweden, grouped by categories of body mass index (BMI).


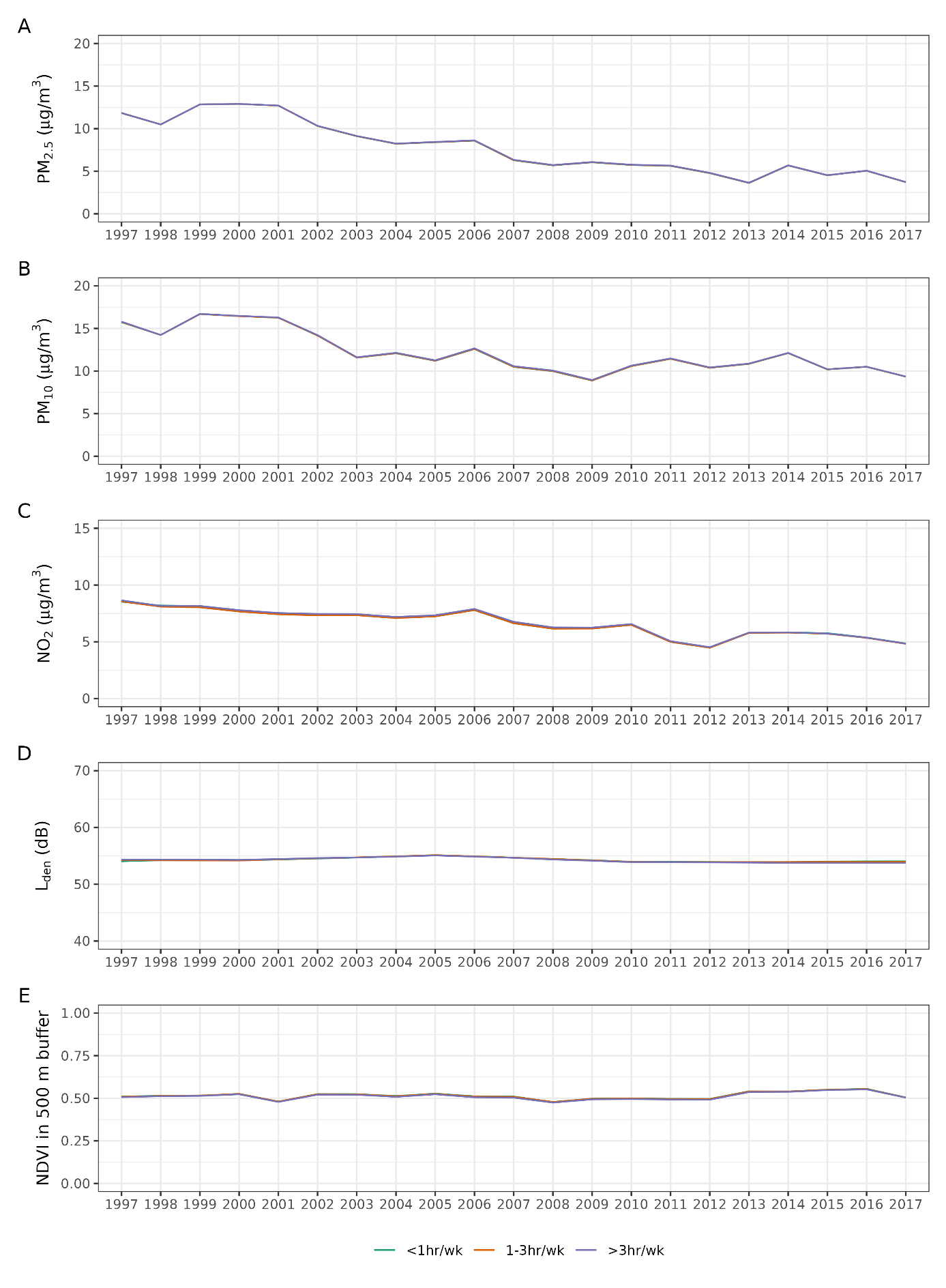


**Figure S14.** **Time-trends of environmental exposures by categories of exercising:** Time-trends of the exposure to A) PM_2,5_, B) PM_10_, C) NO_2_, D) road traffic noise and E) greenness (500 m radius buffer around the residents) among study participants of the Swedish Mammography Cohort (SMC) residing in Uppsala County, Sweden, grouped by categories of time exercising.


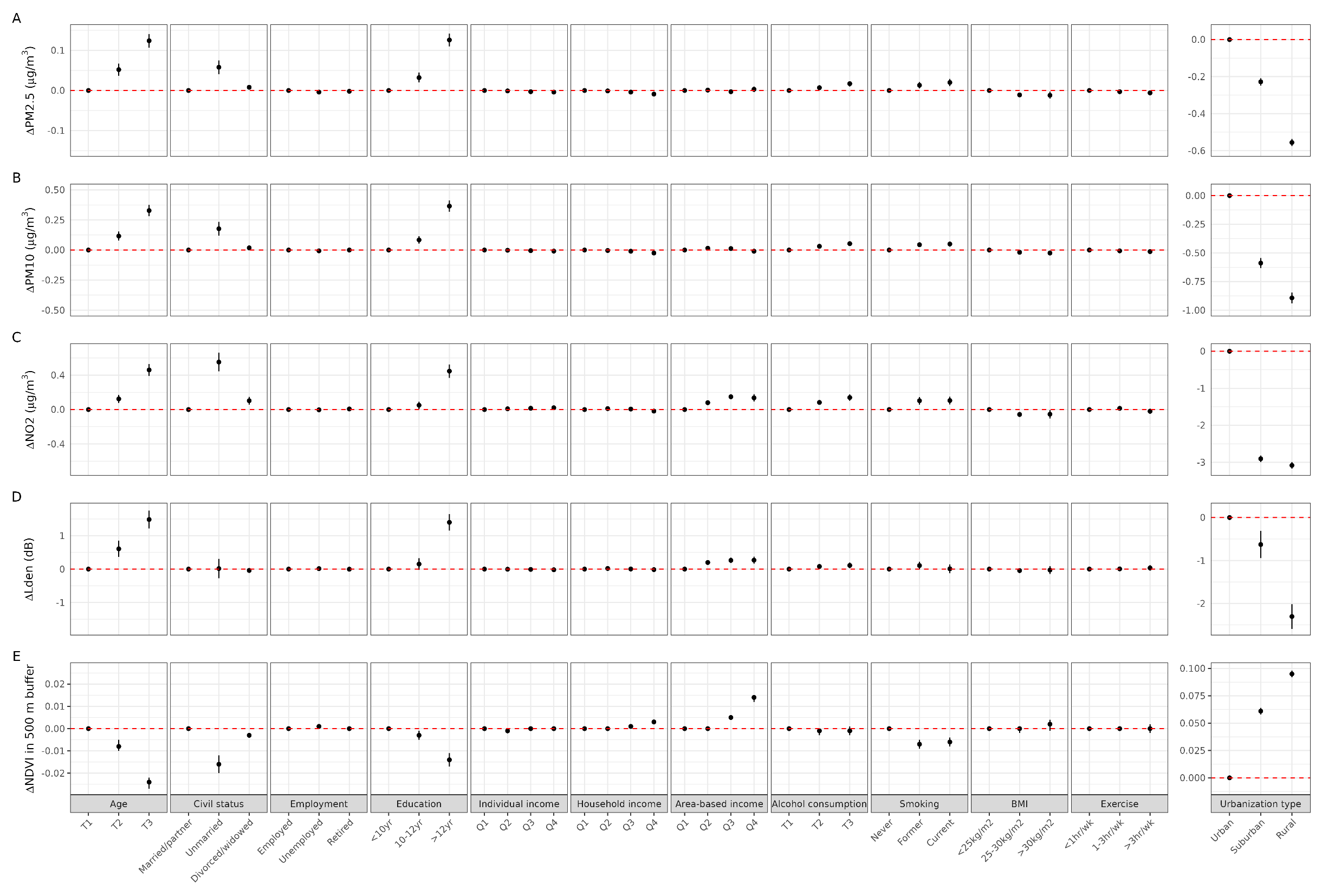

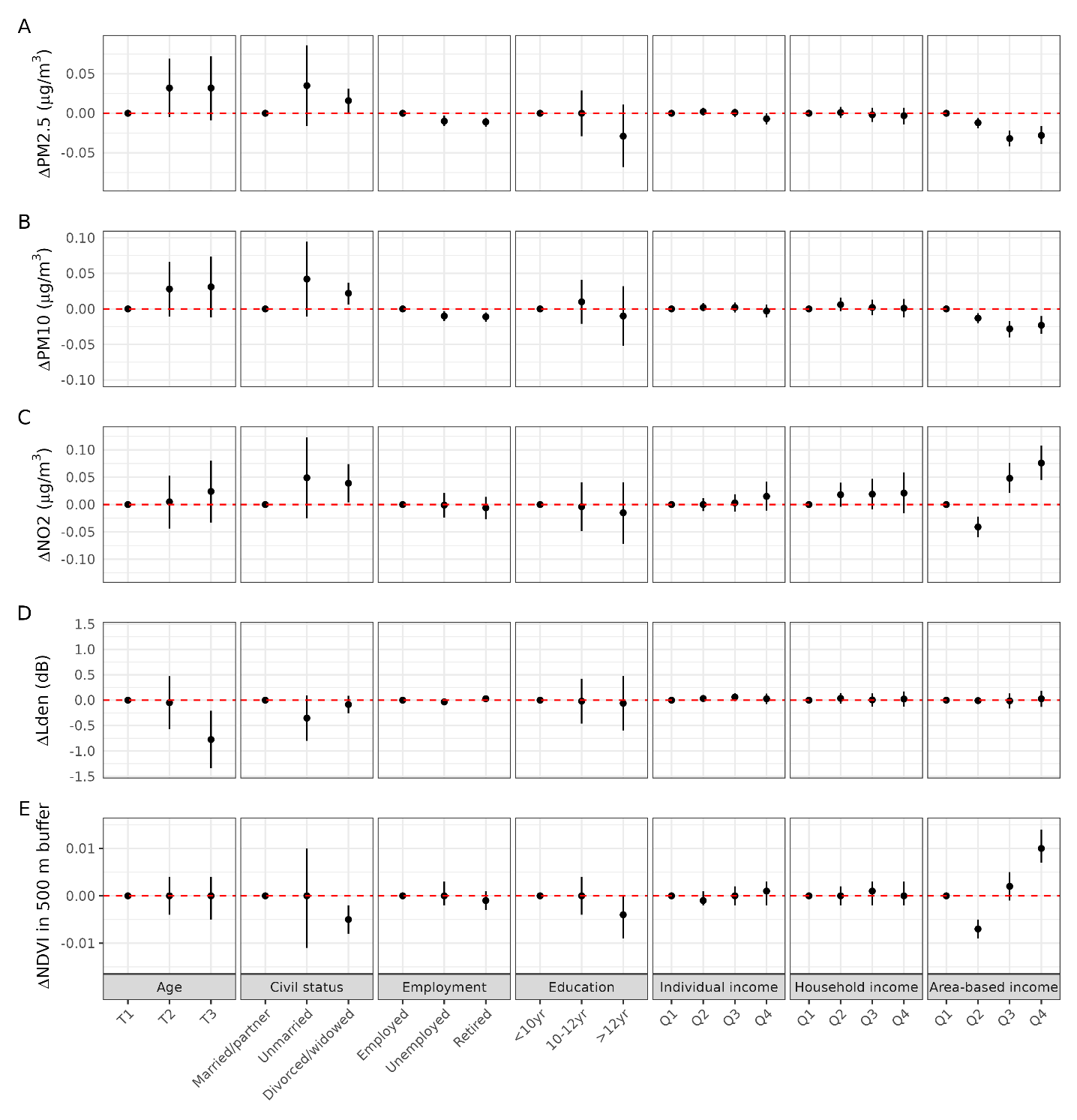


**Figure S15. Sensitivity analysis: Associations between sociodemographic determinants and environmental exposures further adjusted for lifestyle variables.** GEE derived beta-coefficients and 95% confidence intervals (CI) of associations between various socioeconomic determinants and lifestyle covariates, and exposure to air pollution (PM_2.5_, PM_10_, NO_2_), road traffic noise (L_den_), and greenness (NDVI within a buffer of 500 m radius) at the residence adjusted for calendar years. Each row represents one model where the predictors are the sociodemographic and lifestyle factors on the x-axes and the response variables are the environmental exposures on the y-axes. Note the adapted y-scale for the urbanization type in the last column.


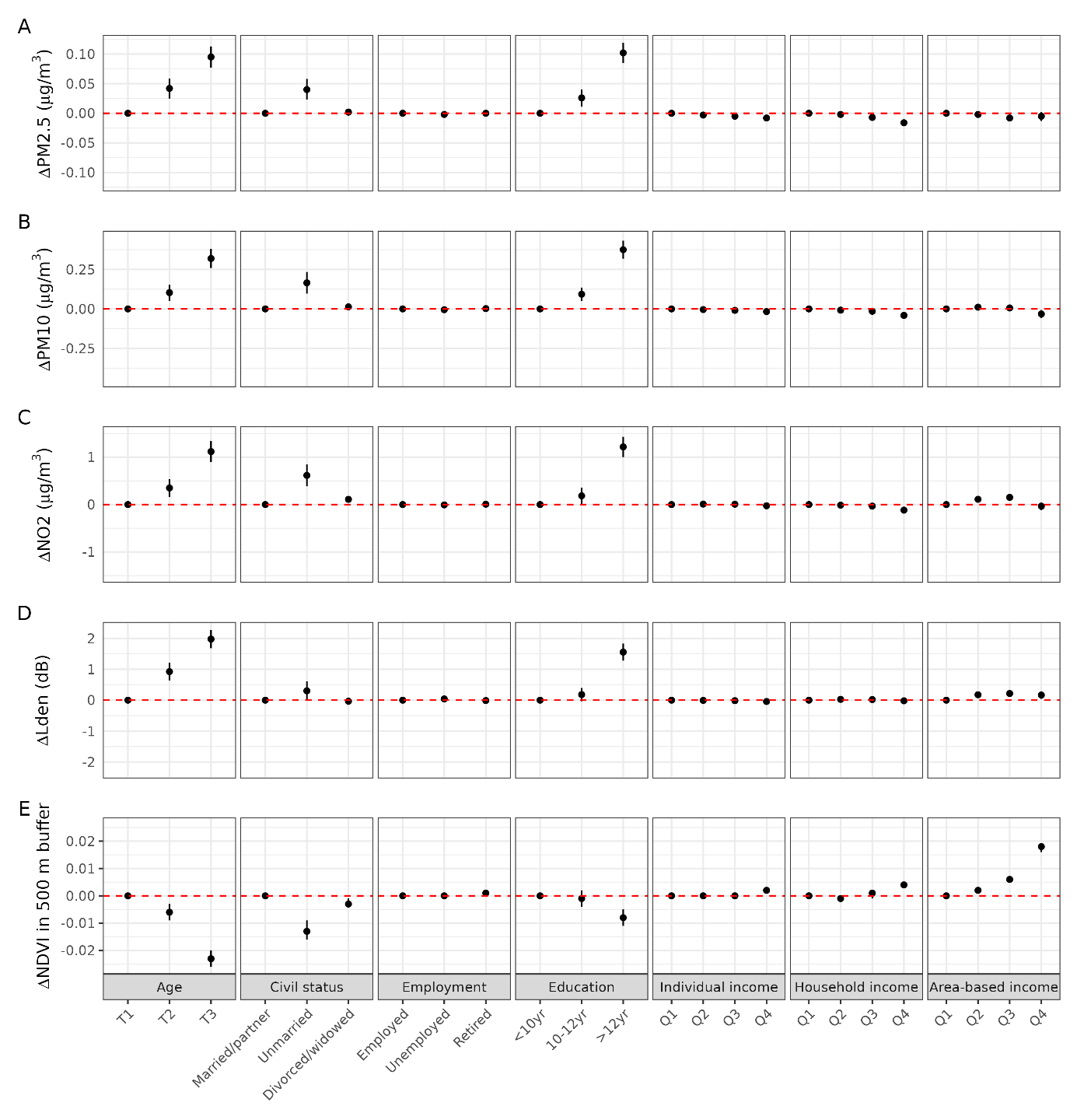


**Figure S16. Stratification: Associations between sociodemographic determinants and environmental exposures restricted to the urban area:** Estimates from the linear regression within GEE to assess the associations between socioeconomic determinant and exposure to air pollution (PM_2.5_, PM_10_, NO_2_), road traffic noise (L_den_), and greenness (NDVI within a buffer with a radius of 500 m) at the residence adjusted for urbanization type and years in the restricted to subpopulation residing in an *urban area*.


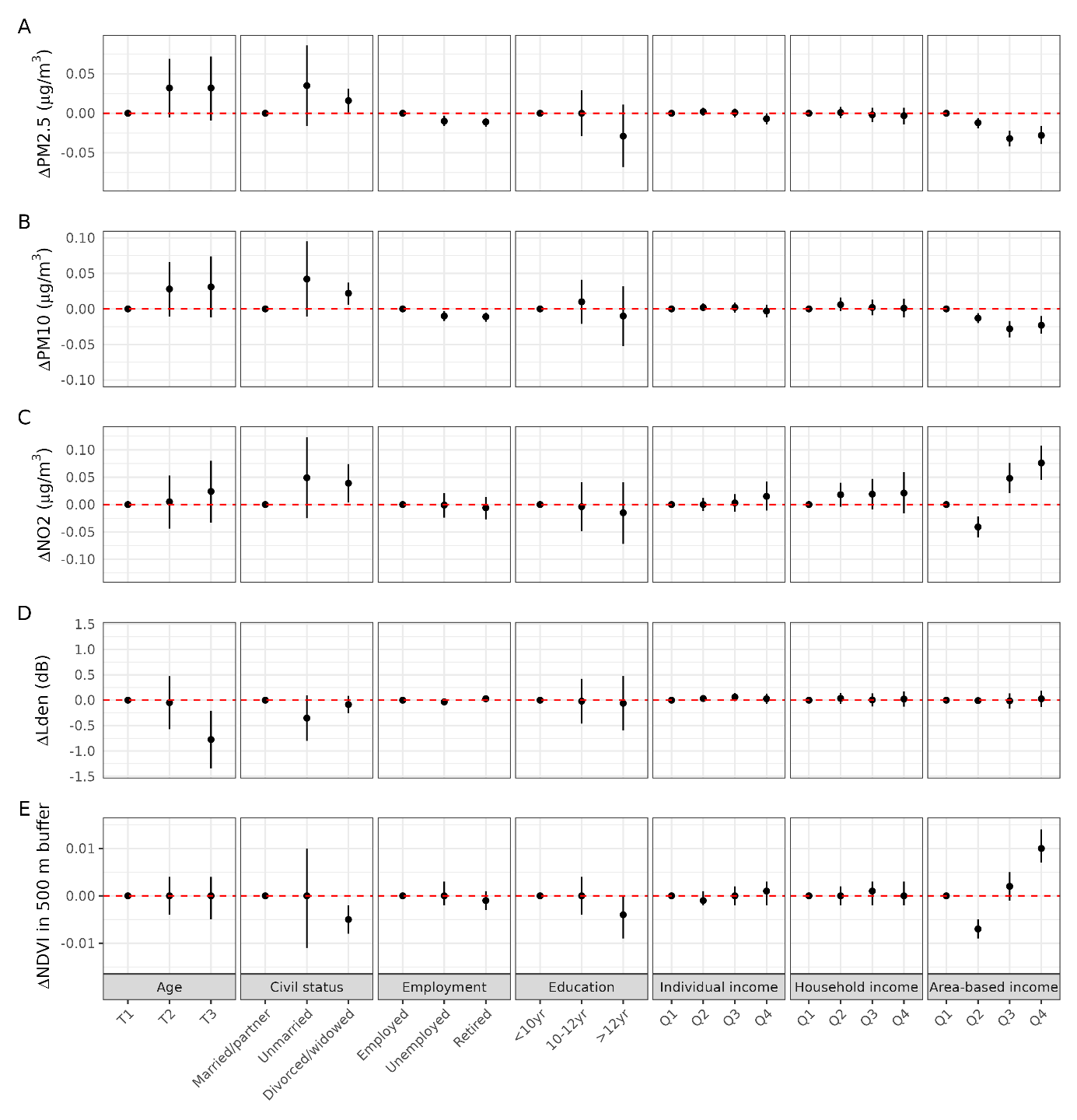


**Figure S17. Stratification: Associations between sociodemographic determinants and environmental exposures restricted to the suburban area:** Estimates from the linear regression within GEE to assess the associations between socioeconomic determinant and exposure to air pollution (PM_2.5_, PM_10_, NO_2_), road traffic noise (L_den_), and greenness (NDVI within a buffer with a radius of 500 m) at the residence adjusted for urbanization type and years in the restricted to subpopulation residing in a *suburban area*.


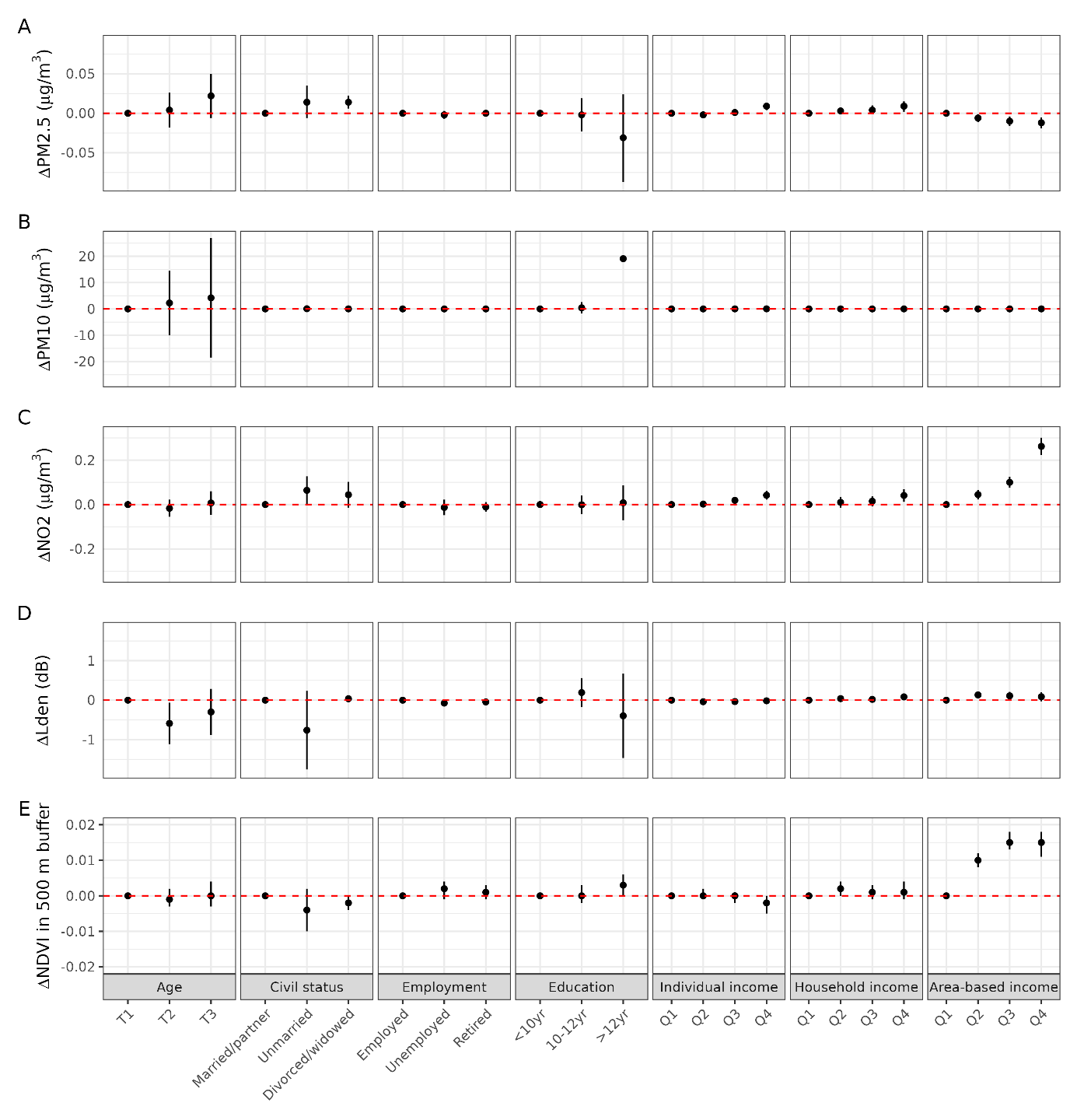


**†**

**Figure S18. Stratification: Associations between sociodemographic determinants and environmental exposures restricted to the rural area:** Estimates from the linear regression within GEE to assess the associations between socioeconomic determinant and exposure to air pollution (PM_2.5_, PM_10_, NO_2_), road traffic noise (L_den_), and greenness (NDVI within a buffer with a radius of 500 m) at the residence adjusted for urbanization type and years in the restricted to subpopulation residing in a *rural area*.

† Beta: 19.08 µg/m^3^, 95 % CI: -84.58 – 122.75

**Table S1.** **Associations between sociodemographic determinants and environmental exposures:** GEE derived beta-coefficients and 95% confidence intervals (CI) of associations between various socioeconomic determinants and exposure to air pollution (PM_2.5_, PM_10_, NO_2_), road traffic noise (L_den_), and greenness (NDVI within a buffer of 500 m radius) at the residence adjusted for calendar years. Each column of environmental exposure and its lower and upper CI represents estimates from one model where the predictors are the sociodemographic factors (rows). The first category is chosen to be the reference category (beta=0). This table complements Figure 3 with numbers.

| **Variable** | **Category** | **PM25** | **PM25 lower** | **PM25 upper** | **PM10** | **PM10 lower** | **PM10 upper** | **NO2** | **NO2 lower** | **NO2 upper** | **Lden** | **Lden lower** | **Lden upper** | **NDVI 500 m** | **NDVI 500 m lower** | **NDVI 500 m upper** |
| --- | --- | --- | --- | --- | --- | --- | --- | --- | --- | --- | --- | --- | --- | --- | --- | --- |
|  | **Intercept** | 11.887 | 11.87 | 11.904 | 15.769 | 15.722 | 15.817 | 8.707 | 8.617 | 8.796 | 53.534 | 53.284 | 53.784 | 0.49 | 0.488 | 0.493 |
| **Age** | **T1** | Ref. | Ref. | Ref. | Ref. | Ref. | Ref. | Ref.0 | Ref.0 | Ref.0 | Ref. | Ref. | Ref. | Ref. | Ref. | Ref. |
| **Age** | **T2** | 0.044 | 0.029 | 0.058 | 0.092 | 0.055 | 0.129 | 0.085 | 0.04 | 0.13 | 0.611 | 0.375 | 0.847 | -0.006 | -0.009 | -0.004 |
| **Age** | **T3** | 0.114 | 0.098 | 0.13 | 0.29 | 0.242 | 0.338 | 0.362 | 0.298 | 0.427 | 1.402 | 1.154 | 1.649 | -0.022 | -0.024 | -0.019 |
| **Civil status** | **Married/partner** | Ref. | Ref. | Ref. | Ref. | Ref. | Ref. | Ref. | Ref. | Ref. | Ref. | Ref. | Ref. | Ref. | Ref. | Ref. |
| **Civil status** | **Unmarried** | 0.061 | 0.045 | 0.078 | 0.18 | 0.127 | 0.233 | 0.52 | 0.419 | 0.621 | 0.038 | -0.253 | 0.329 | -0.016 | -0.02 | -0.012 |
| **Civil status** | **Divorced/widowed** | 0.008 | 0.003 | 0.013 | 0.021 | 0.004 | 0.037 | 0.106 | 0.063 | 0.15 | -0.024 | -0.093 | 0.044 | -0.003 | -0.004 | -0.001 |
| **Employment** | **Employed** | Ref. | Ref. | Ref. | Ref. | Ref. | Ref. | Ref. | Ref. | Ref. | Ref. | Ref. | Ref. | Ref. | Ref. | Ref. |
| **Employment** | **Unemployed** | -0.004 | -0.007 | -0.001 | -0.007 | -0.014 | 0 | -0.007 | -0.029 | 0.014 | 0.012 | -0.029 | 0.053 | 0.001 | 0 | 0.002 |
| **Employment** | **Retired** | -0.002 | -0.005 | 0 | -0.001 | -0.008 | 0.005 | 0.004 | -0.014 | 0.021 | -0.006 | -0.038 | 0.026 | 0 | 0 | 0.001 |
| **Education** | **<10yr** | Ref. | Ref. | Ref. | Ref. | Ref. | Ref. | Ref. | Ref. | Ref. | Ref. | Ref. | Ref. | Ref. | Ref. | Ref. |
| **Education** | **10-12yr** | 0.038 | 0.025 | 0.05 | 0.119 | 0.068 | 0.17 | 0.068 | 0.025 | 0.11 | 0.214 | 0.043 | 0.385 | -0.004 | -0.006 | -0.001 |
| **Education** | **>12yr** | 0.134 | 0.118 | 0.15 | 0.343 | 0.236 | 0.45 | 0.467 | 0.388 | 0.547 | 1.36 | 1.099 | 1.621 | -0.014 | -0.017 | -0.011 |
| **Individual income** | **Q1** | Ref. | Ref. | Ref. | Ref. | Ref. | Ref. | Ref. | Ref. | Ref. | Ref. | Ref. | Ref. | Ref. | Ref. | Ref. |
| **Individual income** | **Q2** | -0.001 | -0.003 | 0.001 | -0.002 | -0.006 | 0.002 | 0.009 | -0.001 | 0.019 | -0.003 | -0.031 | 0.024 | 0 | -0.001 | 0 |
| **Individual income** | **Q3** | -0.002 | -0.005 | 0 | -0.004 | -0.01 | 0.002 | 0.019 | 0.006 | 0.032 | -0.013 | -0.047 | 0.022 | 0 | -0.001 | 0.001 |
| **Individual income** | **Q4** | -0.005 | -0.008 | -0.001 | -0.009 | -0.017 | -0.002 | 0.023 | 0.005 | 0.041 | -0.029 | -0.07 | 0.013 | 0.001 | 0 | 0.002 |
| **Household income** | **Q1** | Ref. | Ref. | Ref. | Ref. | Ref. | Ref. | Ref. | Ref. | Ref. | Ref. | Ref. | Ref. | Ref. | Ref. | Ref. |
| **Household income** | **Q2** | -0.001 | -0.004 | 0.001 | -0.004 | -0.01 | 0.003 | 0.012 | -0.006 | 0.03 | 0.029 | -0.005 | 0.064 | 0 | -0.001 | 0.001 |
| **Household income** | **Q3** | -0.004 | -0.007 | -0.001 | -0.01 | -0.017 | -0.002 | 0.005 | -0.014 | 0.024 | 0.012 | -0.032 | 0.056 | 0.001 | 0 | 0.002 |
| **Household income** | **Q4** | -0.01 | -0.013 | -0.006 | -0.025 | -0.035 | -0.016 | -0.02 | -0.043 | 0.003 | -0.009 | -0.062 | 0.044 | 0.003 | 0.002 | 0.004 |
| **Area-based income** | **Q1** | Ref. | Ref. | Ref. | Ref. | Ref. | Ref. | Ref. | Ref. | Ref. | Ref. | Ref. | Ref. | Ref. | Ref. | Ref. |
| **Area-based income** | **Q2** | 0.001 | -0.002 | 0.005 | 0.017 | 0.01 | 0.025 | 0.084 | 0.066 | 0.103 | 0.2 | 0.148 | 0.252 | 0 | -0.001 | 0.001 |
| **Area-based income** | **Q3** | -0.001 | -0.006 | 0.003 | 0.017 | 0.004 | 0.03 | 0.158 | 0.13 | 0.186 | 0.274 | 0.199 | 0.35 | 0.005 | 0.004 | 0.006 |
| **Area-based income** | **Q4** | 0.004 | -0.002 | 0.01 | -0.004 | -0.023 | 0.015 | 0.148 | 0.106 | 0.19 | 0.273 | 0.175 | 0.371 | 0.014 | 0.012 | 0.015 |
| **Urbanization type** | **Urban** | Ref. | Ref. | Ref. | Ref. | Ref. | Ref. | Ref. | Ref. | Ref. | Ref. | Ref. | Ref. | Ref. | Ref. | Ref. |
| **Urbanization type** | **Suburban** | -0.228 | -0.246 | -0.209 | -0.587 | -0.628 | -0.546 | -2.915 | -3 | -2.83 | -0.67 | -0.964 | -0.376 | 0.059 | 0.056 | 0.062 |
| **Urbanization type** | **Rural** | -0.558 | -0.575 | -0.54 | -0.904 | -0.952 | -0.855 | -3.096 | -3.178 | -3.014 | -2.314 | -2.577 | -2.05 | 0.095 | 0.092 | 0.098 |
